# Resolving distance variations by single-molecule FRET and EPR spectroscopy using rotamer libraries

**DOI:** 10.1016/j.bpj.2021.09.021

**Published:** 2021-09-16

**Authors:** Daniel Klose, Andrea Holla, Christoph Gmeiner, Daniel Nettels, Irina Ritsch, Nadja Bross, Maxim Yulikov, Frédéric H.-T. Allain, Benjamin Schuler, Gunnar Jeschke

**Affiliations:** 1 Department of Chemistry and Applied Biosciences, ETH Zurich, Zurich, Switzerland; 2 Department of Biochemistry, University of Zurich, Zurich, Switzerland; 3 Institute of Biochemistry, ETH Zurich, Zurich, Switzerland; 4 Department of Physics, University of Zurich, Zurich, Switzerland; 5 Department of Chemistry, University of Zurich, Zurich, Switzerland

## Abstract

Förster resonance energy transfer (FRET) and electron paramagnetic resonance (EPR) spectroscopy are complementary techniques for quantifying distances in the nanometer range. Both approaches are commonly employed for probing the conformations and conformational changes of biological macromolecules based on site-directed fluorescent or paramagnetic labeling. FRET can be applied in solution at ambient temperature and thus provides direct access to dynamics, especially if used at the single-molecule level, whereas EPR requires immobilization or work at cryogenic temperatures but provides data that can be more reliably used to extract distance distributions. However, a combined analysis of the complementary data from the two techniques has been complicated by the lack of a common modeling framework. Here, we demonstrate a systematic analysis approach based on rotamer libraries for both FRET and EPR labels to predict distance distributions between two labels from a structural model. Dynamics of the fluorophores within these distance distributions are taken into account by diffusional averaging, which improves the agreement with experiment. Benchmarking this methodology with a series of surface-exposed pairs of sites in a structured protein domain reveals that the lowest resolved distance differences can be as small as ∼0.25 nm for both techniques, with quantitative agreement between experimental and simulated transfer efficiencies within a range of ±0.045. Rotamer library analysis thus establishes a coherent way of treating experimental data from EPR and FRET and provides a basis for integrative structural modeling, including studies of conformational distributions and dynamics of biological macromolecules using both techniques.

## Significance

Combining data from different experimental techniques is often essential for taking advantage of the complementary information they can provide. An area in which this approach has been particularly fruitful is the integrative structural modeling of biological macromolecules, their conformational changes, and their assemblies. Förster resonance energy transfer (FRET) and electron paramagnetic resonance (EPR) spectroscopy are two powerful biophysical techniques that have long been used for this purpose, but integrating them has been complicated by the lack of a suitable analysis framework. Here, we establish such a framework based on rotamer libraries of the labels used in FRET and EPR, and we apply it to a challenging experimental benchmark.

## Introduction

Revealing the detailed functional mechanisms of proteins, nucleic acids, and the biomolecular complexes that they form requires not only information on static structures but also on their conformational distributions and transitions, as well as structural dynamics. Prominent examples include the nuclear pore complex (1,2), protein-RNA complexes involved in splicing regulation (3,4), and G-protein-coupled receptor complexes (5,6). The intrinsic flexibility or structural heterogeneity of these systems is commonly described by ensembles of structures, in which each member contributes with a certain probability. This ensemble forms the basis for a description of the mechanism of action (5,7).

Arriving at such a detailed and complex description is, however, challenging and may require a combination of different techniques. For the structural characterization of a single well-structured state, classical structure determination methods such as X-ray crystallography, NMR spectroscopy, and single-particle cryo-electron microscopy are well established for determining biomolecular structures with atomic resolution (3,8,9). These structures provide the starting point for characterizing transitions to other functional states or conformational heterogeneity and dynamics of mobile elements, such as flexible linkers in multidomain proteins or disordered, functionally relevant regions. In these cases, long-range information on distances and dynamics is particularly important. Long-range distance information can be accessed, for instance, by small-angle scattering, either of X-rays or neutrons (10). These methods offer the advantage—like NMR—that the system is investigated in solution and thus under near-physiological conditions. Although inferring structure from scattering curves is a severely ill-posed problem, small-angle scattering is a valuable tool for providing low-resolution shape information, which has been successfully combined in integrative structure modeling approaches with data from other techniques such as X-ray crystallography, NMR, EPR, FRET, or cryo-electron microscopy (3,4,10, 11, 12, 13, 14, 15, 16, 17, 18, 19).

Another important class of techniques allows for measuring site-selective distances in the low-nanometer range and for characterizing dynamics: electron paramagnetic resonance (EPR) and fluorescence spectroscopy via Förster resonance energy transfer (FRET). Both techniques rely on the site-directed covalent attachment of labels, namely a pair of paramagnetic spin labels or a pair of fluorophores that make the investigated systems EPR or FRET active, respectively. Although FRET is performed in solution at ambient temperature, at which information on dynamics is directly accessible, pulse EPR spectroscopy typically requires low temperatures in the range of 10–50 K, depending on the type of spin label, to reduce longitudinal and transverse relaxation of the electron spins (20, 21, 22, 23, 24, 25).

The distance information accessible from freeze-trapping of different states and the application of pulse EPR spectroscopy is in the range of 1.5–16 nm (20,26,27). Importantly, besides average distances, distance distributions can be obtained that provide information on the width of the structural ensemble of the molecular system (28). The distance distributions between spin labels can be determined by pulse dipolar spectroscopy (PDS), a term that summarizes different methods such as Double Electron-Electron Resonance (DEER) (29,30), RIDME (31), DQC (32), and SIFTER (33). All these techniques measure the distribution of electron dipolar spin-spin coupling, which has an *r*^−3^ distance dependence. The transformation of the time-domain data from PDS experiments to distance distributions is moderately ill posed. Data analysis procedures such as Tikhonov regularization with the L-curve criterion are well established (20,26) and enable the detection of distance distributions that is direct in the sense that the forward calculation of time-domain data from distance distributions is calibration-free because it only depends on fundamental constants. Orientation selection with respect to the dipolar coupling can usually be neglected for solvent-exposed nitroxide spin labels because of their wide conformational distributions. Alternatively, when the orientation between two labels is correlated, the angular information on the relative orientations can be extracted in addition to the distance distribution, albeit with substantial additional effort (20,34,35).

Complementary to EPR, fluorescence spectroscopy offers the advantage of being performed near or at physiological temperatures, at which dynamics can be probed; moreover, it reaches single-molecule sensitivity (36, 37, 38). Distances are most commonly measured by FRET, the nonradiative energy transfer from a donor to an acceptor fluorophore. The rate of energy transfer depends on the coupling between the transition dipole moments of both labels and hence has a distance dependence of *r*^−6^ (39). To minimize the uncertainty from the orientation dependence of the dipole coupling, fluorescence labels typically have linkers that are flexible enough to ensure rapid rotational averaging. Single-molecule fluorescence experiments can be used to probe nanometer distances and conformational dynamics over a wide range of timescales (37,40,41). However, the conformational dynamics are not necessarily separated in time from other contributions such as rotational relaxation, fluorescence lifetimes, and other photophysics, which complicates the analysis in terms of the underlying distance distributions (42, 43, 44, 45).

If long-range distance constraints between fluorescence or spin labels are to be used for structure modeling, it can be essential to explicitly account for the labels in modeling. For spin labels used in pulse EPR spectroscopy, this requirement has led to the development of different molecular modeling approaches. These are either atomistic molecular dynamics (MD) simulations of spin-labeled proteins (23,46, 47, 48) or, alternatively, less computationally demanding molecular models that explicitly take the linker structure and energetics into account. Making use of explicit chemical structure rather than an accessible volume-based approach allows these molecular models to predict the often highly anisotropic conformational distributions of protein-bound nitroxide spin labels. These molecular models, namely rotamer libraries (49,50), MtsslWizzard (51), and ALLNOX (52), have become a standard analysis technique for comparing EPR-derived distance distributions to biomolecular structures (28). Rotamer libraries are a set of precalculated conformers (or rotamers) for each label that are representative of the labels’ conformational space. For any given labeling site in a biomolecule, screening of the libraries allows us to find a set of rotamers that avoids clashes with the protein along with the respective populations for each rotamer that identify energetically preferred states within the labels’ conformational space. This approach has not only been used on single structures but also allows for the rapid analysis of series of structures along normal modes (53,54) or of MD trajectories (55). Simulated distance distributions between two sets of spin-label conformations show an average accuracy of 2.5 Å with respect to experimental distance distributions obtained by DEER (51,56). For integrative structure modeling, the spin-label rotamer libraries have been used successfully together with NMR and crystallographic constraints for modeling flexible protein termini or extended loops and relative positions of flexibly linked domains (57).

For fluorescence labels, a well-established and efficient modeling approach is to calculate the sterically accessible volume (AV) of the dyes (58). The labeled side chain is described by a small number of parameters, such as linker length and width and chromophore radii. Interdye distance distributions or average distances are then calculated from the discretized dye positions within their respective AVs, in which all volume elements are considered with equal weights (59,60). This approach is computationally inexpensive and has been applied in many studies on biomolecules (37,61,62). Rotamer libraries tailored to fluorescence labels have been developed and applied for proteins and nucleic acids (14,63, 64, 65). More detailed molecular models that rely on MD simulations (66, 67, 68, 69, 70) or Monte Carlo sampling (23,71) are computationally more demanding; hence, they lack the efficiency required for analyzing large ensembles of structures and may require a system-specific optimization of force field parameters, especially regarding fluorophore-protein interactions (72).

Using both FRET and EPR to observe biomolecules under different experimental conditions and with or without ensemble averaging yields complementary data on structure and dynamics (11,25). However, directly combining the two methods in integrative structural modeling has been complicated by the lack of a common framework for treating the labels. To overcome this issue, we adopt the approach commonly used in EPR spectroscopy and establish rotamer libraries for FRET, with an optimized force field as a computationally efficient approach for obtaining distance distributions between two protein-linked fluorophores given a model of the structure. For the subsequent calculation of average FRET efficiencies, we account for the label dynamics by treating them in terms of diffusion in a potential of mean force obtained from the distance distributions. To test the methodology, we evaluate the accuracy with which small distance variations can be determined by the two label-based techniques FRET and EPR. For this purpose, we used six double-cysteine variants of a rigid and well-defined protein system consisting of two RNA recognition motifs (RRMs) of the human polypyrimidine tract binding protein 1 (PTBP1) (73, 74, 75). The labeling positions in the two domains (RRM3/4) are shifted by three to seven residues (one or two *α*-helix turns). The single-molecule FRET efficiencies resolved all but one of the distance variations involving a single helix turn. We find that taking into account the motion of the fluorophores improves the accuracy of the absolute distances recovered. For EPR spectroscopy, the resulting distance distributions between spin labels were well suited for revealing both the absolute distances and the relative distance shifts by one or two helix turns for five out of six variants. Hence, both techniques can detect distance variations with a precision corresponding to one *α*-helix turn. The common framework of rotamer library simulations establishes a way of combining experimental data from FRET and EPR spectroscopies in a unified approach suitable for integrative modeling.

## Materials and methods

All chemicals were purchased from Sigma-Aldrich (Buchs, Switzerland), unless stated otherwise.

### Labeling sites and protein expression

The two-domain construct RRM3/4 of PTBP1 represents a well-structured and conformationally rigid system (74). For distance measurements between spin labels or fluorescence labels, we selected positions separated by distances in a range of 1.5–8.0 nm. As described earlier, the site pair Q388C-S475C delivered narrow distance distributions when investigated with a set of different spin labels (75). Based on this site pair, we selected solvent-exposed *α*-helical sites separated by four to seven amino acids from Q388C and S475C, respectively, which translates to one or two helix turns. In total, five positions were chosen for mutagenesis, two on RRM3 (Q388C and S392C) and three on RRM4 (E468C, V472C, and S475C). The insertion of cysteine residues at the respective positions was achieved by site-directed mutagenesis, and the constructs of RRM3/4 bearing double-cysteine mutations were expressed and purified as previously described (75).

### Spin labeling

The different mutated and purified (75) proteins were spin-labeled with a 10-fold excess of 3-maleimido proxyl (MAP) at ambient temperature. The labeling reaction was performed overnight in labeling buffer (50 mM 3-(N-morpholino)propanesulfonic acid (MOPS), 25 mM NaCl (pH 6.5)) under gentle shaking. Residual unbound spin label was removed by washing the samples with a low-salt buffer (10 mM NaPO_4_, 20 mM NaCl (pH 6.5)) via PD10 desalting columns (GE Healthcare, Glattbrugg, Switzerland). Eluted proteins were concentrated to a final volume of ∼200 *μ*L with a concentration of 100 *μ*M. Sample quality was checked by SDS-PAGE under nonreducing conditions and labeling efficiencies were determined by continuous wave (cw) EPR spectroscopy.

### Fluorescence labeling

For site-specific labeling with Cy3b (GE Healthcare, Little Chalfont, UK) and CF660R (Sigma-Aldrich, St. Louis, MO), the double-cysteine constructs of RRM3/4 were reduced with 10 mM dithiothreitol (DTT) and further purified by cation exchange chromatography using a 1-mL MonoS column (GE Healthcare; 20 mM KPO_3_/PO_4_, 10% glycerol (pH 7.2), gradient: 0–500 mM KCl). The freshly reduced protein was incubated with CF660R maleimide overnight at 4°C at a molar ratio of dye:protein of 0.7:1 (76). Cation exchange chromatography was used to enrich unlabeled and single- and double-labeled species after quenching the reaction with 10 mM DTT. In most cases, these labeling reaction products eluted from the column in four peaks, with some peaks partially overlapping, namely the double-labeled species with one labeling permutant and the other labeling permutant with the unlabeled species. Nevertheless, the labeling permutants with the label attached at either of the cysteines eluted in clearly separated peaks, thus allowing site-specific labeling of RRM3/4. Single-labeled RRM3/4 was subsequently incubated with an excess of Cy3b maleimide overnight at 4°C to label the second cysteine. Free label was removed by cation exchange chromatography after quenching the reaction with 10 mM DTT. Because attaching Cy3b did not cause a significant shift in elution of the protein from the column, this purification step did not lead to further separation of donor-acceptor-labeled protein from the double-donor- or double-acceptor-labeled species, so that depending on the labeling permutant, the resulting material contains either a small amount of double-donor- or double-acceptor-labeled species in addition to the donor-acceptor-labeled species. In two cases, CF660R maleimide reacted only with one of the cysteine residues in the first labeling step. To produce both permutants of those variants, the labeling procedure had to be reversed. In those cases, Cy3b maleimide was added to the unlabeled protein first, followed by CF660R maleimide in the second labeling step. With this procedure, the dyes were site-specifically attached at the two labeling positions, resulting in 12 donor-acceptor-labeled variants altogether, including two permutants per construct. The attachment positions of the dyes were identified by trypsin digest followed by mass spectrometry.

### EPR spectroscopy

For cw EPR experiments, 20 *μ*L of sample with a protein concentration of ∼25 *μ*M were transferred into glass capillaries with 0.9 mm outer diameter (Blaubrand micropipettes; Brand, Wertheim, Germany). cw EPR measurements were carried out in the X band (∼9.5 GHz) on an Elexsys E500 EPR spectrometer (Bruker Biospin, Rheinstetten, Germany) equipped with a Bruker super-high-Q resonator at room temperature. Spectra were recorded with 100 kHz magnetic field modulation, 0.1 mT modulation amplitude, and a lock-in time constant and conversion time of 10.24 and 40.96 ms, respectively. The power was attenuated by 25 dB of 200 mW incident microwave power. The spin labeling efficiency was determined by digital double integration of the cw EPR spectra using a reference solution of 100 *μ*M 3-(2-iodoacetamido)-proxyl and comparing to the protein concentration.

For pulse EPR experiments, protein samples were first diluted with D_2_O to a concentration of ∼75 *μ*M and then mixed with d_8_-glycerol in a ratio of 1:1. 30 *μ*L sample was added to 3 mm quartz capillaries (Aachener Quartzglas, Aachen, Germany) and shock frozen in liquid nitrogen.

Pulse EPR experiments were carried out at Q-band frequencies (∼34.5 GHz) using a home-built spectrometer (77), equipped with a traveling wave tube amplifier with 200 W nominal microwave power, as well as a home-built TE102 dielectric resonator (78) and a helium flow cryostat (Oxford Instruments, Oxfordshire, UK) to stabilize the temperature to 50 K. DEER data were acquired using the four-pulse DEER sequence (30), *π*/2 − *τ*_1_ − *π* − (*τ*_1_ + *t*) − *π*_*pump*_ − (*τ*_2_ − *t*) − *π* − *τ*_2_ − *echo*, by incrementing the pump pulse delay *t* in steps of 12 or 16 ns (for *τ*_2_ > 4 *μ*s) with a two-step phase cycle of the first *π*/2 pulse to cancel receiver offsets. All pulse lengths were set to 12 ns, and the offset between pump and observer frequencies was set to 100 MHz, with the pump pulse positioned at the maximum of the nitroxide spectrum (78). Nuclear modulation was averaged out by stepping *τ*_1_ in eight steps of 16 ns starting from *τ*_1_ = 400 ns (33,78). The second delay time *τ*_2_ was set between 4 and 7 *μ*s according to the expected distances, and the dead-time delay was 280 ns.

The resulting time traces were analyzed in DEERAnalysis 2016 (79) using a 3D-homogeneous background function. Subsequently, distance distributions were obtained from the model-free Tikhonov regularization (80,81) with the regularization parameter determined according to the L-curve criterion (82).

### Single-molecule FRET measurements

For single-molecule experiments, the donor-acceptor-labeled protein variants were diluted to 100 pM in 20 mM Tris, 125 mM KCl (pH 7.4) with 0.001% Tween 20, 10 mM DTT, and 5 nM unlabeled RRM3/4 as additives. The experiments were conducted at 22°C using chambered cover slides (*μ*-Slide; ibidi, Gräfelfing, Germany) on a custom-built confocal instrument described previously (83), equipped with a supercontinuum source (SuperK EXTREME EXW-12; NKT Photonics, Birkerød, Denmark) to excite the donor dye and a 635-nm diode laser operated in pulsed mode (LDHD-C-635M; PicoQuant, Berlin, Germany) to excite the acceptor dye. Both lasers were operated at a pulse repetition rate of 20 MHz. The light from the NKT source was filtered with a bandpass filter (BrightLine HC 520/5; Semrock, Rochester, NY). Fluorescence photons were collected through a high-numerical-aperture objective (UPlanApo 60×/1.20-W; Olympus, Tokyo, Japan), subsequently separated from the scattered photons with a triple-band mirror (zt405/530/630rpc; Chroma, Bellows Falls, VT), and distributed onto four channels according to their wavelength and polarization. Dichroic mirrors were used to separate donor and acceptor emission (T635LPXR; Chroma). Donor photons were selected with ET585/65m bandpass filters (Chroma) before detection on one of two single-photon avalanche photodiodes (*τ*-SPAD, PicoQuant). Acceptor photons were selected with LP647RU longpass filters (Chroma) and detected with SPCM-AQRH-14 single-photon avalanche photodiodes (PerkinElmer, Waltham, MA). To remove the contribution of molecules with inactive acceptor, pulsed interleaved excitation was used (84). Time bins of 1 ms containing more than 50 photons (after background correction) emitted upon donor or acceptor excitation were regarded as photon bursts corresponding to a single protein diffusing through the confocal volume. Photon counts were corrected for background, differences in quantum yields of the dyes, different detection efficiencies, spectral cross talk, and direct excitation of the acceptor dye by the donor excitation light. The necessary correction factors were inferred from the measurement of a set of calibration samples (45) labeled with Cy3b and CF660R following the procedure given in (45,85,86). For each burst, the transfer efficiency *E* = $N_{A}^{d}/\left(N_{A}^{d}+N_{D}^{d}\right)$ and the stoichiometry ratio *S* = $\left(N_{A}^{d}+N_{D}^{d}\right)/\left(N_{A}^{d}+N_{D}^{d}+N_{A}^{a}\right)$ were calculated, where $N_{A}^{d}$ and $N_{D}^{d}$ are the corrected numbers of acceptor and donor photons emitted upon donor excitation and $N_{A}^{a}$ the corrected numbers of acceptor photons emitted upon acceptor excitation. The mean values ⟨E⟩ and ⟨S⟩ of subpopulations were determined via 2D-Gaussian fits to 2D *S* vs. *E* histograms (see Fig. S3). Based on the polarization sensitivity of the four-channel detection system, fluorescence anisotropies were quantified for all samples from the fluorescence emission of the donor- and acceptor-only subpopulations. Because the resulting steady-state anisotropies for all samples were below 0.12, we assume for our analysis that the rotational correlation time of the dyes is sufficiently short for a rotationally averaged orientation factor of 2/3 in Förster theory (86,87).

The FRET pair Cy3b/CF660R was chosen in part because of its relatively low sensitivity to changes in local environment upon labeling (45), which is supported by the low root mean-square deviation of 0.016 from *S* = 0.5 averaged over all variants, with no individual deviation being greater than 0.02. Moreover, the fluorescence lifetimes exhibited only a small variability among protein labeling variants. The average donor lifetime of all variants (obtained from the donor-only populations) was 2.85 ns, with a standard deviation of 0.08 ns, close to the estimated uncertainty of the donor lifetime measurements of 0.06 ns. The average acceptor lifetime of all variants (obtained by direct excitation of the acceptor) was 3.24 ns, with a standard deviation of 0.05 ns, close to the estimated uncertainty of the acceptor lifetime measurements of 0.03 ns. Moreover, we observe no correlation between the deviations from *S* and the fluorescence lifetimes of the individual variants, indicating that the small residual deviations from *S* = 0.5 for individual variants cannot be accounted for by differences in quantum yields reflected by altered fluorescence lifetimes. Possible contributions may originate from residual static quenching, which can affect observed transfer efficiencies without affecting fluorescence lifetimes (45,88).

### Rotamer library generation and simulation of distance distributions

Rotamer libraries contain precalculated preferred conformations of the spin or fluorescence label of interest. The precalculation is carried out once for a new label and allows for generating a rotamer library that can subsequently be integrated into the open-source software-tool Multiscale Modeling of Macromolecules (MMM) (57), available on www.epr.ethz.ch and on GitHub.com/gjeschke. Once such a library is available, rotamer library analysis (RLA) and calculation of distance distributions can be carried out in MMM for any biomolecular structure (details below).

The generation of a rotamer library consists of three steps: geometry optimization, Monte Carlo sampling, and clustering.

#### Geometry optimization

The labeled side chains of cysteine-maleimido-CF660R or cysteine-maleimido-Cy3b were first drawn in 3D using the open-source molecular editor Avogadro 1.2.0 (89), which also provided an initial geometry optimization using the universal force field (UFF) (90). Further geometry optimization was carried out by density functional theory in Orca 3 (91) at the restricted Kohn-Sham level of theory using the functional BP86 (92,93) and the triple-*ζ* basis set def2-TZVP (94), with the resolution of identity (RI) approximation (95,96). Density functional theory calculations further included the conductor-like screening model (97,98), a continuum model to mimic bulk electrostatics of the solvent water.

#### Monte Carlo sampling

The total conformational space of the labeled side chains is in good approximation given by combining all possible torsion states of the rotatable dihedral angles, of which there are 8 and 12 for Cy3b and CF660R, respectively. Hence, choosing the dihedral angles as the only degrees of freedom, we used a variant of Metropolis Monte Carlo sampling (99) to generate ensembles of 500,000 structures representative of all side-chain conformations using custom-written scripts in MATLAB 2018b (The MathWorks, Natick, MA). The energies taken into account are the dihedral angle and Lennard-Jones potentials from the UFF force field (90) with parameters from the Towhee implementation (100). The Lennard-Jones potential is of the form

(1) $$E_{LJ}=\sum_{i,j}\varepsilon_{ij}\left[{\left(\frac{f\sigma_{ij}}{r_{ij}}\right)}^{12}-2{\left(\frac{f\sigma_{ij}}{r_{ij}}\right)}^{6}\right],$$

where *E*_*LJ*_ is the total Lennard-Jones energy summed over all pairs of atoms with indices *i* and *j*. ε_*ij*_ and *σ*_*ij*_ are the depth of the potential well and the van der Waals radius, respectively, specific to atoms *i* and *j* as taken from from the UFF force field. The *f* factor, also known as the “forgive factor,” is an empirical softening parameter for the potential that we used for small spin labels only when computing the interaction of the label with the protein (50). A different modified Lennard-Jones potential was used in construction of rotamer libraries for native amino acid side chains (101). For chromophores as well as for large spin labels, it is important to soften the potential also for intralabel atom pairs, when only dihedral degrees of freedom are considered, to account for the combined effects of small bond and angle variations that sum up with the increasing length of a label. The *f* factor also allows us to tune the attractive term in the potential in the absence of a solvent model, whereas a cutoff distance was used for native side chains in SCWRL4 (101). Because there is currently no method available to predict an optimal *f* value, rotamer libraries with different *f* factors were compared to the experimental data. Based on this comparison (vide infra), we used *f* = 0.175 unless stated otherwise. This value is much lower than the ones used previously for generating rotamer libraries of small spin labels (*f* = 0.7…1.0) or for computing spin label-protein interactions (*f* = 0.4…0.6).

For Monte Carlo sampling, random values for the dihedral angles are drawn from a set of values distributed according to the dihedral potentials. The Lennard-Jones energy *ε*_*i*_ of the new conformer is calculated and Boltzmann-weighted with a temperature *T* = 298 K and the Boltzmann constant *k*_*B*_ to generate a population $p_{i}^{MC}$ = exp(−(*ε*_*i*_ − *ε*_0_)/(*k*_*B*_*T*)) with respect to a minimal Lennard-Jones energy *ε*_0_ determined before by 5 × 10^7^ Monte Carlo trials. If the normalized population $p_{i}^{MC}$ is ≥1%, the new conformer is accepted into the resulting Monte Carlo ensemble. The threshold of 1% ensures sufficient sampling of the large number of canonical rotamers. The ensemble size of 500,000 used here is at the limit of what the subsequent clustering step could process in our case. Sampling appears sufficiently converged at this size for both labels, as judged from the dihedral-angle histograms (Fig. S6).

#### Clustering

The resulting Monte Carlo ensembles were reduced to a smaller number of *N*_*rot*_ structures by hierarchical clustering using custom-written MATLAB scripts, where *N*_*rot*_ should be large enough to sufficiently represent the full conformational space of the label and small enough for computational efficiency. The clustering algorithm calculates the pairwise similarity of all members of the Monte Carlo ensemble using distance in dihedral angle space as the metric. This procedure divides the initial ensemble into *N*_*rot*_ clusters of similar ensemble members. For each cluster, one average structure was determined to represent the whole group in the final library of *N*_*rot*_ rotamers. The average was taken in torsion angle space to avoid unphysical conformers. For each rotamer, its population $p_{i}^{rot}$ was calculated as $p_{i}^{rot}=\sum_{j}p_{j}^{MC}$ by summing up the populations *p*_*j*_ of the cluster members, which results in normalized probabilities with $\sum_{i}p_{i}^{rot}$ = 1.

#### Simulation of distance distributions

Simulations of the spatial distribution of the individual spin or fluorescence labels as well as of the distance distributions were carried out using MMM (57). First, loading a structure file, we used either all 20 structures of the solution NMR ensemble (Protein Data Bank (PDB): 2ADC (74)) or, when stated, only the first NMR structure with truncation of the flexible N-terminus (RRM3/4-ΔN without residues G324 to N336). In both cases, only the protein in the structure file was considered. Then, all labeling sites (residues) and one label, such as *MAP* in the case of the spin label maleimido proxyl, were selected. For each site, the populations *p*_*i*_ for all *i* rotamers are calculated as *p*_*i*_ = $p_{i}^{clash}\times p_{i}^{rot}$, where $p_{i}^{clash}$ is the *i*-th rotamer population due to clashes of the label with other residues of the protein. The clash energies $\varepsilon_{i}^{clash}$ were determined by the Lennard-Jones potential as above. Subsequent Boltzmann weighting yields populations $p_{i}^{clash}=\text{exp}\left(-\varepsilon_{i}^{clash}/k_{B}T\right)$/*Z*_*clash*_ with *T* = 298 K and *Z*_*clash*_ = ∑_*j*_exp$\left(-\varepsilon_{j}^{clash}/k_{B}T\right)$ such that $\sum_{i}p_{i}^{clash}=1$. Finally, we also normalize *p*_*i*_ to ∑_*i*_*p*_*i*_ = 1.

For each pair of labeling sites, distance distributions were then calculated as population-weighted histograms of all pairwise distances *r*_*ij*_ between populated rotamers on the two sites *i* and *j*, i.e., a histogram of *p*_*i*_ × *p*_*j*_ × *r*_*ij*_ for all pairs {*i*, *j*}. For the ensemble distance distributions, we considered only site pairs within the same conformer. Hence, for each of the 20 structures, a distance distribution was calculated, and subsequently these distributions were summed up and renormalized to ∑_*r*_*P*(*r*) = 1 to form the final ensemble distribution.

The rotamer libraries for the fluorescence labels maleimido-Cy3b and maleimido-CF660R bound to cysteine (called *Cy3* and *CF6*, respectively, in MMM) were made available as custom rotamer libraries under the names Cy3_298K_UFF_N_r1 and CF6_298K_UFF_N_r1, where N is the library size with 1024, 2048, 4096, or 8192 rotamers (see Supporting materials and methods for further details). The distance *r*_*ij*_ between two fluorescence labels was approximated by the distance between the centers of the two chromophores using the midpoint between the central oxygen and the opposite carbon atom. After selection of the rotamer libraries, the same workflow (see Supporting materials and methods) was used as for spin labels.

### Calculation of mean transfer efficiencies from interdye distance dynamics in a potential of mean force

From peak fitting of the measured transfer efficiency histograms, we obtained the mean transfer efficiencies ⟨E⟩. For comparing these measured values with the theoretical values derived from a distance distribution *P*(*r*), we need to take into account that the interdye distance *r* fluctuates on a timescale similar to the fluorescence lifetime of the donor (2.7 ns). Hence, we model the interdye dynamics in terms of diffusive motion in a potential of mean force corresponding to *P*(*r*), as recently described (102). Briefly, we used the general relation (103)

(2) $$\left\langle E\right\rangle =1-\left\langle \tau^{\text{∗}}\right\rangle /\tau_{D},$$

where *τ*_*D*_ = 1/*k*_*D*_ is the mean fluorescence lifetime of the donor in the absence of an acceptor and $\left\langle \tau^{\text{∗}}\right\rangle =\overset{\infty }{\underset{0}{\int }}p_{D^{\text{∗}}}$(*t*)*dt* is the mean lifetime of the excited state of the donor dye in the presence of the acceptor. *p*_*D*^∗^_(*t*) is the survival probability of the excited state, which we calculate as *p*_*D*^∗^_(*t*) = $\overset{\infty }{\underset{0}{\int }}$*p*_*D*^∗^_(*r*, *t*)*dr* (103) from the solution of the Smoluchowski equation:

(3) $$\frac{\partial p_{D^{\text{∗}}}\left(r,t\right)}{\partial t}=\left[L-k_{D}-k_{D}{\left(\frac{R_{0}}{r}\right)}^{6}\right]p_{D^{\text{∗}}}\left(r,t\right)$$

with the diffusion operator

(4) $$L=D\frac{\partial }{\partial r}P\left(r\right)\frac{\partial }{\partial r}{\left(P\left(r\right)\right)}^{-1},$$

where *D* is the effective diffusion coefficient for the relative translational motion of the fluorophores attached to the protein, given by the sum of the diffusion coefficients of the individual attached dyes. The Smoluchowski equation needs to be solved with the initial condition *p*_*D*^∗^_(*r*, *t* = 0) = *P*(*r*), assuming that the donor was excited at *t* = 0 and that the average interphoton time is much longer than the relaxation time of the interdye distance.

For the calculations, we discretized the Smoluchowski equation with respect to *r* over an interval (*r*_*a*_, *r*_*b*_) outside which *P*(*r*) is zero to good approximation. As a result, Eq. 3 becomes a rate equation:

(5) $$\frac{d}{dt}p^{*}=\left(K^{\text{diff}}+K^{\text{depop}}\right)p^{*},$$

for the vector **p**^∗^ of components $p_{i}^{\text{∗}}$(*t*), with *i* = 1…*N* indicating the discretization steps. The *N* × *N* matrix **K**^diff^ describes the dye diffusion and contains the following nonzero matrix elements:

(6) $$K_{i,i\pm 1}^{\text{diff}}=\frac{D}{\delta r^{2}}\frac{P\left(r_{i\pm 1/2}\right)}{P\left(r_{i\pm 1}\right)}\text{ and }K_{i,i}^{\text{diff}}=-K_{i-1,i}^{\text{diff}}-K_{i+1,i}^{\text{diff}},$$

where *r*_*i*_ = *r*_*a*_ + (*i* − 1)*δr* with *δr* = (*r*_*b*_ − *r*_*a*_)/(*N* − 1). The matrix **K**^depop^ is diagonal with elements

(7) $$K_{i,i}^{\text{depop}}=-k_{D}\left(1+{\left(R_{0}/r_{i}\right)}^{6}\right)$$

and describes the depopulation of the excited donor state. The mean lifetime of the excited state of the donor dye is then calculated from

(8) $$\left\langle \tau^{\text{∗}}\right\rangle =\int_{0}^{\infty }1^{T}e^{\left(K^{\text{diff}}+K^{\text{depop}}\right)t}p_{eq}dt,$$

where **1**^*T*^ = (1, 1, …) is the transposed vector of ones, **p**_*eq*_ is the vector defined by **K**^diff^**p**_0_ = 0, and **1**^*T*^**p**_0_ = 1. The effective diffusion coefficient *D* is difficult to measure. Here, we use recently published all-atom MD simulations of polyproline-11 labeled at its ends with Alexa 594 and Alexa 488 (72) to obtain an interdye distance distribution *P*(*r*) and the time correlation of the distance (see Fig. S4), which we fit with (104)

(9) $$G\left(\tau \right)=\frac{1^{T}R\;e^{K^{\text{diff}}\tau }\;Rp_{0}}{{\left(Rp_{0}\right)}^{2}},$$

where *D* is the only fit parameter. **R** is the diagonal matrix with elements **R**_*ii*_ = *r*_*i*_. All calculations were done with *N* = 50. We used *τ*_*D*_ = 2.7 ns (105) for Cy3b (derived from the subpopulation with a stoichiometry ratio of one, i.e., the population corresponding to molecules lacking an active acceptor). The Förster radius for the Cy3b/CF660R dye pair, *R*_0_ = 6.0 nm, was calculated using the emission spectrum of Cy3b, the absorption spectrum of CF660R (both of the free dye), a fluorescence quantum yield of 0.67 for Cy3b, an excitation coefficient of 100,000 M^−1^ cm^−1^ for CF660R (manufacturer’s specifications), a refractive index of 1.334 (100 pM in 20 mM Tris, 125 mM KCl (pH 7.4) with 0.001% Tween 20), and *κ*^2^ = 2/3.

## Results

**Figure 1:**
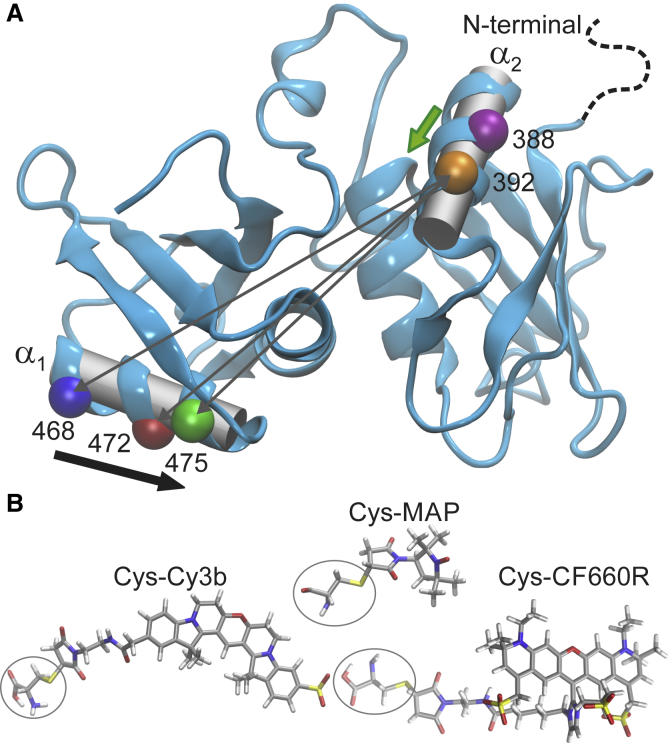
Cysteine positions for fluorescence and spin labeling. (*A*) PTBP1-RRM3/4 (*blue ribbons*) with labeling positions highlighted (*C*_*α*_ atoms as *colored spheres*, connected by *gray arrows* for S392C). Positions are offset by single turns along the two helices *α*_1_ and *α*_2_ (*gray cylinders*), resulting in a shift to smaller distance (*black arrow*) from E468C (*blue*) to S475C (*green*), or from Q388C (*magenta*) to S392C (*orange*) (*green arrow*). The flexible N-terminus is indicated by a dashed line. (*B*) Fluorescence- and spin-labeled cysteine side chains (*stick representation*) are drawn to scale with respect to (*A*). To see this figure in color, go online.

To probe both absolute distances and small distance variations, we designed six double-cysteine variants of a protein construct containing the two domains RRM3 and RRM4 of PTBP1 (RRM3/4), which provides a well-structured scaffold for positioning the labels (74,75). In each variant, one cysteine is located on *α*-helix 1 of RRM4 (*α*_1_) and the other on *α*-helix 2 on RRM3 (*α*_2_, see Fig. 1). On *α*_1_, one of the following residues are replaced by cysteine: E468 (*blue*), V472 (*red*), or S475 (*green*); on *α*_2_, one of two positions: Q388 (*magenta*) or S392 (*orange*). In this way, the neighboring label positions are separated by one helix turn, which allows us to probe small yet well-defined differences in intramolecular distances by comparing these labeling positions (Fig. 1 *A*). The selected residues are located at solvent-exposed sites that allow for accessibility and sufficient steric freedom of the labels. The described choice of positions is based on recently published EPR distance distribution measurements (Q388C-S475C) (75). We labeled the six double-cysteine variants of RRM3/4 either with pairs of identical spin labels for DEER distance distribution measurements or with donor-acceptor dye pairs for single-molecule FRET measurements, using maleimide-based coupling chemistry in both cases. Fig. 1 *B* shows the chemical structures of the labels including their linkers, the maleimide group, and the cysteine. (The representation is drawn to scale with respect to the structure of RRM3/4 in Fig. 1 *A*.) The linker of the nitroxide spin label MAP is just long enough to reduce interference with the protein structure; the fluorescence labels, Cy3b and CF660R, have longer linkers to reduce fluorescence quenching and provide the rotational flexibility required for averaging the orientation factor to *κ*^2^ = 2/3 (36). The structure of Cy3b has been published (106), and the structure of CF660R was determined based on a patent (107) and experimental analysis (see Supporting materials and methods).

**Figure 2:**
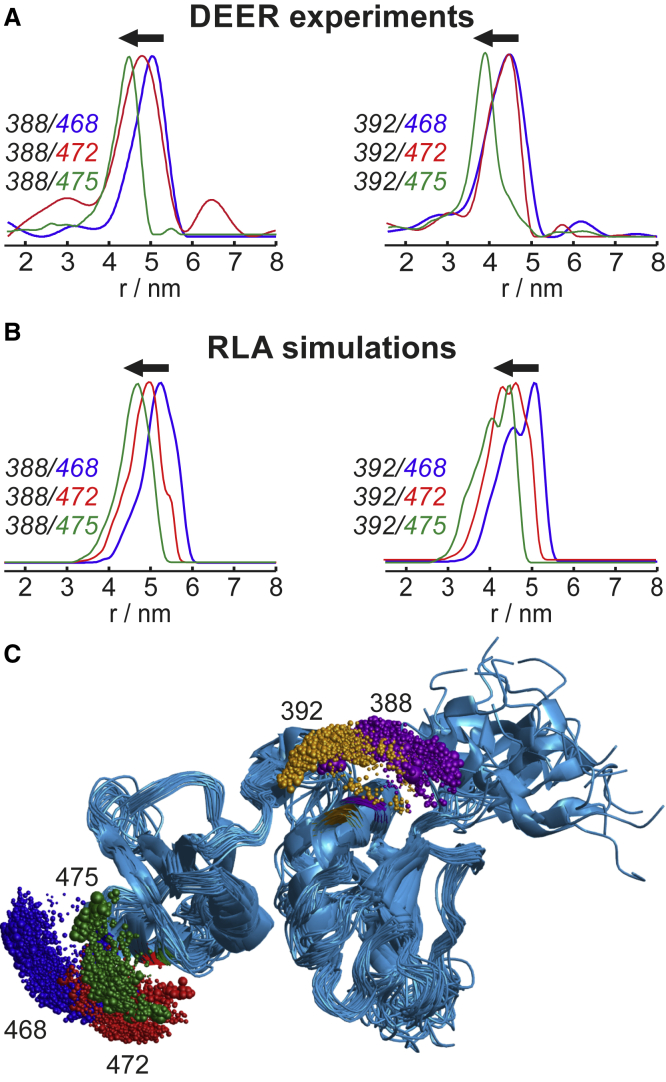
EPR distance determination by DEER and simulations. (*A*) Experimental DEER distance distributions (Fig. S2 shows primary data) between RRM3/4 cysteine positions as indicated in the legend, labeled with maleimido proxyl. (*B*) Rotamer library simulations for maleimido proxyl for all 20 conformations in the NMR ensemble (PDB: 2ADC (74)). Arrows (in *A* and *B*) indicate the shift to smaller distances from E468C (*blue*) to S475C (*green*). (*C*) NMR ensemble of RRM3/4 (*blue ribbons*, PDB: 2ADC (74)) with point clouds indicating the conformational distributions of maleimido proxyl-labeled side chains from the RLA simulations (*colored spheres* indicate label positions, size indicates population). Distances between the point clouds are distributed as shown in (*B*). To see this figure in color, go online.

Successful spin labeling was verified by cw EPR spectroscopy (Fig. S1); the labeling efficiencies are given in Table S1. Distance distributions between spin labels measured by four-pulse DEER (30) with subsequent analysis by model-free Tikhonov regularization (80,81) are shown in Fig. 2 *A* (see Fig. S2 for time-domain data) and report on distances and, because the signal/noise ratio achieved here is sufficient, also on distribution widths between the N-O groups present in the sample. The widths of the distance distributions arise from the conformational distributions of both the protein and the spin-labeled side chains. These are the conformations present at the glass transition temperature of the matrix (108), which are trapped upon rapid freezing. In case of the rather rigid RRM3/4 (75), the width of the distance distribution is dominated by the conformational distribution of the spin labels. Accordingly, we model the distance distributions by RLA, in which the protein structure is kept fixed, and all label conformations are considered that do not clash with the protein, computed using a softened Lennard-Jones potential as described in Materials and methods. The distance distributions resulting from these RLA simulations for all 20 RRM3/4 conformations available in the NMR structure ensemble (PDB: 2ADC (74)) are shown in Fig. 2 *B*. The conformational distributions of the spin labels, visualized in Fig. 2 *C*, illustrate the different states occupied with different probabilities (*sphere sizes*) that give rise to the anisotropic conformational space of the spin labels, as well as the partial spatial overlap of conformations at adjacent labeling positions. However, the center of gravity is clearly shifted when comparing labels at the different positions. This behavior is also reflected quantitatively by the simulated distance distributions (Fig. 2 *B*); they overlap, yet their centers are visibly shifted for all positions if one label is moved by a single helix turn (cf. Table S1).

The experimental distance distributions show the same trend as the simulations. The three distributions in which position 388 is labeled on *α*_2_ are clearly shifted to larger distances compared to the corresponding distributions in which position 392 is labeled. The predicted distance shifts for positions 468 → 472 → 475 are also observed in the DEER distance distributions, with the exception of 468 → 472 if position 392 is labeled. In this case, the measured distributions overlap almost completely, and mean distances differ by only 0.1 nm. The similarity of the DEER time-domain data (Fig. S2) underlines that spin labels at these two positions exhibit very similar distances. Taken together, the comparison between the DEER distance distributions and RLA simulations demonstrates that, in most cases, PDS is sensitive enough to measure distance differences as small as a shift of the labeling position by a single helix turn. In a few cases, the difference is obscured by the conformational distribution of the spin labels, an effect whose contribution is expected to depend on the relative orientation of the two *α*-helices. In terms of absolute distances, we find good agreement to within 2.2 Å on average (Table S1) between the experimental and simulated distance distributions, similar to the 2.5 Å reported for other nitroxide spin labels (51,56).

**Figure 3:**
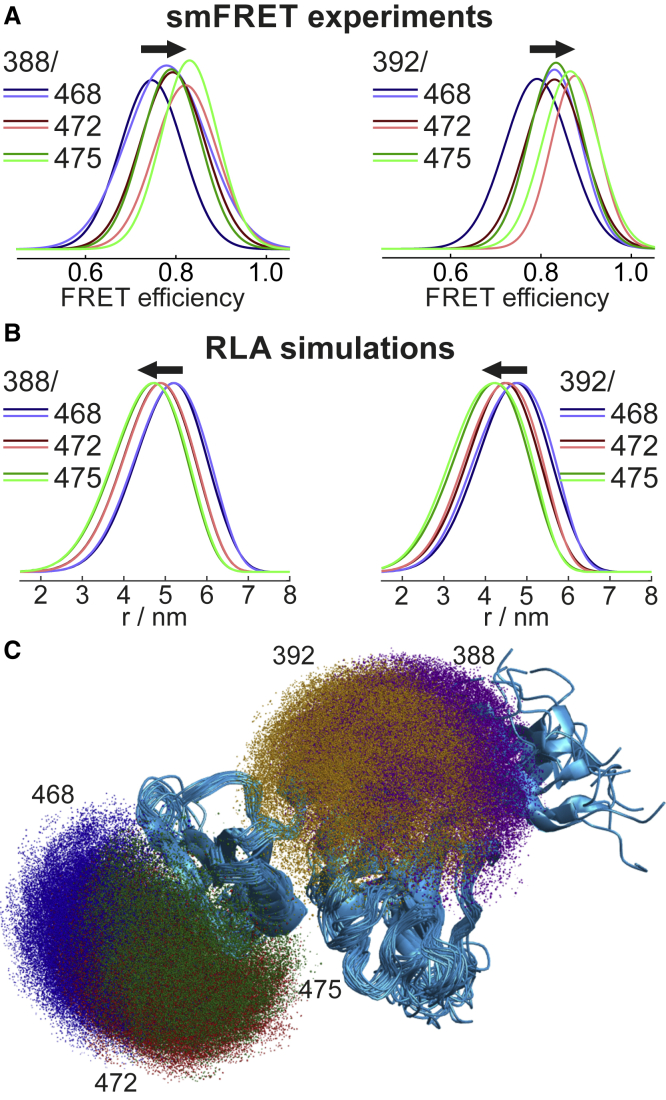
Single-molecule FRET measurements and comparison with simulations. (*A*) Experimental single-molecule FRET efficiency histograms for RRM3/4 represented by the Gaussian fits of the peaks (*solid lines*; cf. Fig. S3 for the original histograms, which are omitted here to improve visualization of the small differences). The labeling positions are indicated (see legend), with darker or lighter colors for helix *α*_2_ labeled with the acceptor or the donor dye, respectively. (*B*) Interdye distance distributions obtained from RLA simulations for all 20 conformations of the NMR ensemble (PDB: 2ADC (74)) with the same color code as in (*A*). Arrows in (*A*) and (*B*) indicate the shift to smaller distances or higher FRET efficiencies from E468C (*blue*) to S475C (*green*). (*C*) NMR ensemble of RRM3/4 (*blue ribbons*, PDB: 2ADC (74)) with spatial distributions of the chromophore centers (*point clouds*) from rotamer library simulations for fluorescence label Cy3b on Q388C and S392C and for CF660R on E468C, V472C, and S475C for all 20 conformations in the NMR ensemble. To see this figure in color, go online.

EPR distance measurements are carried out between identical spin labels, but distance measurements based on FRET require a pair of different fluorescence labels. We employed substoichiometric labeling with the first dye, followed by chromatographic separation of RRM3/4 singly labeled at one cysteine or the other and subsequent labeling of the corresponding other site with the second label (see Materials and methods for details). This approach led to site-specific labeling of RRM3/4 with the donor Cy3b and the acceptor CF660R and allowed for separate measurements of both label permutations for each pair of sites. The resulting single-molecule FRET efficiency histograms of all 12 RRM3/4 labeling variants, measured in free diffusion at 22°C, are shown in Figs. 3 *A* and S3 (Fig. 3 shows only the peak functions used to fit the histograms to make the small differences in peak positions visible). Note that the widths of the peaks in the FRET efficiency histograms are dominated by shot-noise broadening owing to the limited number of photons detected while single protein molecules diffuse through the confocal volume (109, 110, 111). Because the diffusion time through the focus is in the millisecond range but the interdye distance dynamics occur on the nanosecond timescale, only the mean transfer efficiencies ⟨E⟩ are obtained from the peak positions in the histograms (see Table S3). The transfer efficiencies clearly shift to higher values when the *α*_2_ labeling positions are moved from 388 to 392 and also for *α*_1_ positions 468 → 472, 475, whereas for 472 → 475 we observe a clear shift only for two out of four RRM3/4 variants. Notably, ⟨E⟩ for the donor-acceptor labeling permutations on the same double-cysteine constructs reveals small but significant differences, with consistently lower ⟨E⟩ if CF660R is attached at positions 388 or 392. Static acceptor quenching by surface residues close to helix *α*_2_ might contribute to the observed differences (88) (Table S3). We note that the average difference in ⟨E⟩ between the permutants of 0.039 is in a similar range as the uncertainty in ⟨E⟩ from a recent multilaboratory benchmark study (86) and would result in a difference in inferred distance of ∼0.2 nm.

For comparing the experimental ⟨E⟩ with the structure-based predictions, we first calculate distance distributions between the fluorophores and subsequently use these to predict FRET efficiencies, taking into account the dynamics of the flexible linkers. To calculate distance distributions, we generated rotamer libraries for the fluorescence labels by extending our previous approach for spin labels (50) to significantly larger sampling and clustering capacities required for the larger fluorophores (see Materials and methods for details). The libraries ideally cover all label conformations allowed by dihedral rotations. Depending on the local symmetry, the potentials of the dihedral angles have multiple minima, the canonical dihedral angles. The total number of possible label conformations is given by the product of the numbers of minima of all dihedral angles. Cysteine labeled with the common nixtroxide methanethiosulfonate spin label has five dihedral angles, *χ*_1_ to *χ*_5_, which have 3,3,2,3,4-fold symmetry and hence give rise to 3 × 3 × 2 × 3 × 4 = 216 canonical conformers (50); cysteine labeled with MAP has five rotatable dihedral angles, with 3,3,3,1,1-fold symmetry (27 canonical rotamers). In addition, for the MAP library, we had considered two diastereomeric pairs of enantiomers with equal probability and had hence ended up with 108 conformers. This is in stark contrast to the fluorescence labels, which have many more dihedral angles, namely 8 and 12 for Cy3b and CF660R, respectively, leading to 26,244 and 2,125,764 canonical conformers for the two labels. This illustrates how the number of configurations grows exponentially with the number of dihedral angles, which is a challenge both for sampling and for representing these by increasingly large rotamer libraries.

Accordingly, to generate the rotamer libraries for the two fluorescence labels Cy3b and CF660R (see Fig. S5 for flowchart), we started from structures of the fluorophore-labeled cysteines that were geometry-optimized using quantum chemistry calculations (see Materials and methods for details). Subsequently, we generated conformational ensembles with 500,000 structures of the free labels using parallel Monte Carlo sampling in dihedral angle space applying a softened Lennard-Jones potential combined with the dihedral angle potentials provided by the UFF force field (90,100). The *f* factor tunes two effects: it accounts for otherwise neglected flexibility due to small variations in bond lengths and angles that accumulate with increasing chain length, and it adjusts the attractive term of the label-protein potential to model effective surface interactions. Because the optimal *f* factor is not known a priori, we scanned a range of values (see Materials and methods) for comparison to the experimental data. The resulting Monte Carlo ensembles were checked for convergence (Fig. S6), clustered in dihedral angle space (Figs. S7 and S8), and we checked the clustering (Fig. S9) to find a reduced subset of representative rotamers. This set of representative conformers forms a rotamer library.

The advantage of precalculating rotamer libraries is that subsequent calculations of energy-weighted label conformations at a specific site on any protein become computationally much less expensive and hence more readily accessible compared to Monte Carlo sampling of the label conformations directly on the protein. These RLA simulations of RRM3/4 labeled at different positions with Cy3b and CF660R give rise to distance distributions calculated between the centers of the two chromophores (Fig. 3 *B*). The distance distributions calculated with differently softened Lennard-Jones potentials reveal a nonlinear dependence of distances and distribution widths on the *f* factor (Fig. S10) because of a balance of the two effects it tunes, which underlines the importance of validating the model against experimental data (see below). The volumes over which the chromophore centers are distributed on RRM3/4 are shown in Fig. 3 *C* and illustrate both the extent of the spatial delocalization due to the linkers and the rather smooth and isotropic nature of the conformational space accessible to the fluorescence labels compared to the spin labels with their shorter linkers (Fig. 2 *C*), which renders the rotamer libraries of Cy3b and CF660R less sensitive to the library size (Fig. S10). Distributions of the orientation factor *κ*^2^ (72) for the different label pairs yield average *κ*^2^-values of 0.644–0.674 (data not shown), close to the ideal isotropic value of *κ*^2^ = 2/3, and the extent of the accessible conformational space gives rise to distance distributions between Cy3b and CF660R that are between 1.99 and 2.25 nm wide (full width at half maximum (FWHM)) (Table S2). Despite this considerable width, the mean values are clearly shifted to shorter distances along the series 468 → 472 → 475 in *α*_1_ as well as when comparing *α*_2_ positions 388 → 392 for all three positions of the second label (Fig. 3 *B*). When exchanging the donor and acceptor labels in the simulations, we do not observe significant changes in the distance distributions for any of these combinations (Fig. 3 *B*; Table S2).

For comparing with the experimental FRET results, we need to calculate the expected mean transfer efficiencies from the simulated interdye distance distributions. This procedure is, however, complicated by the relative diffusive motion of the dyes, which occurs on the same timescale (nanoseconds) as the excited state lifetime of the donor (43,44,72). This contribution is ignored when assuming a static distance distribution and calculating the mean transfer efficiency from ⟨E⟩=∫*E*(*r*)*P*(*r*)*dr*, where *E*(*r*) is the transfer efficiency at the (instantaneous) interdye distance *r* (42, 43, 44,102,112). Furthermore, we assume rotational diffusion to be faster than translational diffusion (63) and accordingly *κ*^2^ averaging to be fast. We thus account for the dynamics by describing the fluctuations in interdye distance in terms of diffusive motion in a potential of mean force, with the potential calculated from the distance distribution obtained by the RLA simulations (see Materials and methods for details). In this diffusional averaging procedure, the largest uncertainty originates from the assumed effective diffusion coefficient *D*, which is difficult to measure. An upper limit is given by *D* = *D*_1_ + *D*_2_, where *D*_1_ and *D*_2_ are the translational diffusion coefficients of the free dyes. The diffusion coefficient of Cy3b at room temperature in water is 0.44 nm^2^/ns (105). Assuming this value for both dyes results in ∼0.88 nm^2^/ns as an upper limit for *D*. However, the dyes’ motion is expected to be slowed down when they are attached to the protein. Peulen et al. (44) recently obtained a diffusion coefficient of Alexa488-C5-maleimide attached to the human guanylate binding protein 1 of 0.1 nm^2^/ns by comparing simulated fluorescence decays for various diffusion coefficients with the corresponding experimental fluorescence decay. The simulated decays were obtained from Brownian dynamics simulations of the dye in its AV and included quenching of the dye upon collision with amino acids on the protein surface. Assuming the same diffusion coefficient for both dyes used here would result in *D* = 0.2 nm^2^/ns. We obtained a very similar value from recently published all-atom MD simulations of polyproline-11 labeled with Alexa594 via an N-terminal Gly and with Alexa488 via a C-terminal Cys (72). Using the interdye distance distribution and time correlation derived from the MD simulations, we determined an effective diffusion coefficient of *D* = 0.22 nm^2^/ns (see Materials and methods and Fig. S4 for details). We are not aware of such values for the specific combination of Cy3b and CF660R, but in view of the similarity in fluorophore size to Alexa488/594, we assume *D* ≈ 0.2 nm^2^/ns.

**Figure 4:**
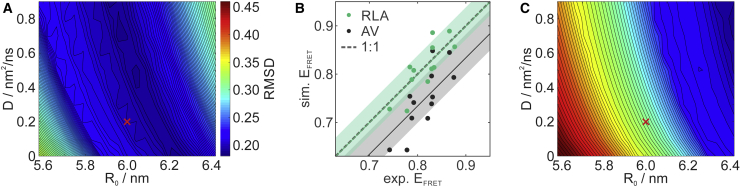
Comparison of experimental smFRET efficiencies to predictions using diffusional averaging over distance distributions. (*A* and *C*) RMSD between experimental and simulated FRET efficiencies averaged over all 12 data sets (see Table S3) for different Förster radii (*R*_0_ ± 7% (86)) and effective diffusion constants of the fluorescence labels based on the distance distributions (see Figs. 3 and S12) of the RLA simulations (*A*) and AV simulations (*C*) using the same color scale given in (*A*). The red crosses mark *R*_0_ = 6.0 nm and *D* = 0.2 nm^2^/ns and correspond to the circles in (*B*). (*B*) Experimental versus simulated FRET efficiencies from RLA (*green*) and AV analysis (*black*) with shaded bands corresponding to uncertainties in Förster radius *R*_0_ and effective diffusion constant *D* (see *A*). Linear fits to the data with slope 1 (*green* and *black lines* for RLA and AV, respectively) show deviations from the ideal 1:1 correlation (*gray dashed line*). To see this figure in color, go online.

The Förster radius, too, can only be determined to limited accuracy, with a recent estimate of its uncertainty of ∼7% (86). Because of the described uncertainties in *D* and *R*_0_, the systematic uncertainties for the transfer efficiencies $\langle E\rangle_{\text{sim}}$ calculated from the RLA distributions are much greater than the statistical errors in the measured transfer efficiencies $\langle E\rangle_{\text{exp}}$ (less than ±0.01; see Table S3). To visualize the effect of the uncertainties in *D* and *R*_0_ on $\langle E\rangle_{\text{sim}}$, we thus show in the contour plot of Fig. 4 *A* the root mean-square deviation (RMSD) between $\langle E\rangle_{\text{sim}}$ and $\langle E\rangle_{\text{exp}}$ averaged over all 12 RRM3/4 variants (including labeling permutations) calculated for *R*_0_ ranging from 5.6 to 6.4 nm and *D* ranging from 0 (no diffusion) to 0.9 nm^2^/ns (free-dye diffusion). The combination of values we consider most likely according to the discussion above, *R*_0_ = 6.0 nm and *D* = 0.2 nm^2^/ns, is indicated with a red cross. The simulated values $\langle E\rangle_{\text{sim}}$ from diffusional averaging, calculated for these parameters (*R*_0_ = 6.0 nm and *D* = 0.2 nm^2^/ns) are compared to the experimental values $\langle E\rangle_{\text{exp}}$ in Fig. 4 *B* (*green*) for all 12 RRM3/4 variants. The shaded bands reflect the uncertainty in *R*_0_ and *D* that are calculated as standard deviations of all transfer efficiencies for the *R*_0_, *D* combinations shown in Fig. 4 *A*, i.e., for *R*_0_ = 6.0 nm ± 7% and *D* = 0…0.9 nm^2^/ns for all 12 RRM3/4 variants. The comparison of simulated and experimental transfer efficiencies allows us to determine the optimal Lennard-Jones softening *f* factor, because for plausible values of *R*_0_ and *D*, agreement between simulated and experimental FRET efficiencies is obtained only in a narrow range for the *f* factor, around *f* = 0.175 (Fig. S11). For this value, the standard deviation owing to *R*_0_ and *D* overlaps with the ideal 1:1 correlation, and the RMSD shows a clear minimum and thus underlines the optimal choice of *f* for this particular set of rotamer libraries for Cy3b and CF660R (Fig. S11). For *f* factors outside the range of *f* = 0.15–0.2, the discrepancy between $\langle E\rangle_{\text{exp}}$ and $\langle E\rangle_{\text{sim}}$ cannot be compensated by varying *R*_0_ and *D* in reasonable ranges. Hence, we consider the decrease in RMSD around this interval a significant improvement. Note that the optimal *f* factor for the fluorescence labels is smaller than the values of *f* = 1.0…0.7 usually applied to generate rotamer libraries of spin labels. This finding points to two different regimes: one for the short spin labels, in which particular conformations of the linker dominate, and one for the longer fluorophore linkers, in which a coarse-grained model with populations assigned per unit volume rather than per rotamer may become a suitable representation for the spatial distribution. This interpretation also explains the decreased sensitivity of the distance distributions we observed for rotamer library sizes smaller than the number of canonical rotamers for the fluorescence labels (Fig. S10). For the optimal rotamer libraries, we found that the lowest RMSD between experimental and predicted FRET efficiencies is close to the most likely values of *R*_0_ = 6.0 nm and *D* = 0.2 nm^2^/ns. Using these parameters for the modeling of FRET efficiencies by diffusional averaging, we find agreement with the experimental results to within ±0.025 in half of the cases and to within ±0.045 in 11 out of our 12 cases (Table S3).

For comparison, we also calculated the distance distributions resulting from AV simulations (37,58) for the N-terminal truncation variant RRM3/4-ΔN (Fig. S12). We found the single RRM3/4-ΔN structure to be a suitable representation for the ensemble of all 20 NMR structures, as indicated by the similarity of the resulting RLA distance distributions in both cases (Fig. S13; Table S2). In analogy to Fig. 4 *A*, we also calculated, for the distance distributions derived from the AV analysis, the RMSD to the experimental transfer efficiencies as a function of *R*_0_ and *D* (Fig. 4 *C*). The result shows that a larger Förster radius of *R*_0_ ≈ 6.3 nm and/or a higher value for *D* would need to be assumed to reach similarly low RMSD values as in the RLA simulations. Nevertheless, simulated transfer efficiencies based on the AV distance distributions that include diffusional averaging with *D* = 0.2 nm^2^/ns (see Fig. 4 *B*, *black*) are closer to the experimental values than transfer efficiencies based on the static FRET approximation (see Fig. S11; Table S3). Notably, AV-based transfer efficiencies calculated both with and without diffusional averaging showed systematically lower values than the experiment. Hence, the prediction closest to the experimental results for our test set of 12 RRM3/4 variants are the transfer efficiencies calculated with diffusional averaging using the RLA-derived distance distributions (Figs. 4 *B* and S11; Table S3), suggesting that the spatial distributions of the fluorophores are better represented by the rotamer distributions using this optimization (Fig. 3 *C*) than by the AV distributions with parameters derived from the label structure (Fig. S12).

**Figure 5:**
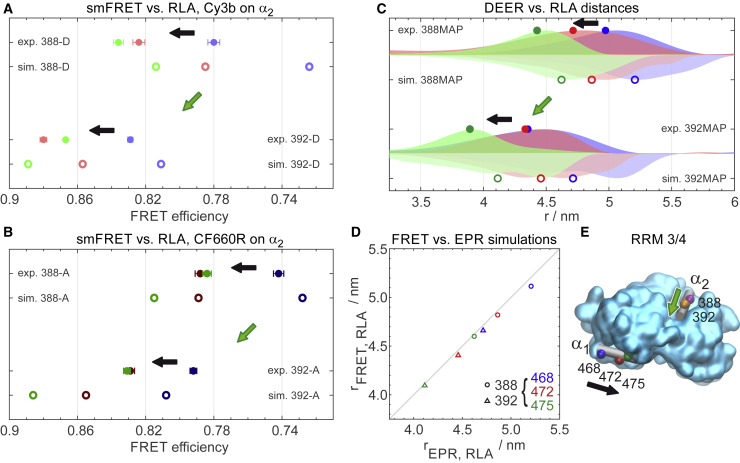
Resolving distances and distance variations by EPR and single-molecule FRET (smFRET) using RLA simulations. (*A*) Comparison of experimental FRET efficiencies (*filled symbols*, error bars represent standard deviations estimated from multiple measurements (Table S3)) with simulated results using RLA distance distributions and diffusional averaging (*open symbols*) for positions 388 and 392 on helix *α*_2_ labeled with Cy3b with the second label at positions 468, 472, and 475 in blue, red, or green, respectively. (*B*) is analogous to (*A*) but for CF660R attached to *α*_2_. (*C*) Comparison of DEER and RLA distance distributions oriented upwards or downwards, respectively. Labeling positions along *α*_2_ are indicated, and positions 468, 472, and 475 on *α*_1_ are shown in blue, red, or green, respectively. The symbols indicate the average distances. (*D*) Correlation between RLA average distances calculated for EPR and FRET (see Tables S1–S3 for data). (*E*) Labeling positions on RRM3/4 with arrows, which indicate distance variations between positions along *α*_1_ (*black*) or *α*_2_ (*green*) in all panels. To see this figure in color, go online.

Our observations from both EPR and single-molecule FRET are summarized in Fig. 5. Shifting the labeling position on helix *α*_2_ from 388 to 392 reduces the distance to helix *α*_1_ and thus leads to increased transfer efficiencies, as calculated using diffusional averaging and the RLA distributions (see Fig. 5, *green arrows*). The same behavior is observed in the experiments for all three positions on *α*_2_ and all corresponding label permutations (Fig. 5*, A and B*, *green arrows*). Similarly, shifting the labels from position 468 on *α*_1_ by a single helix turn to position 472 (see Fig. 5, *black arrows*) results in consistent shifts in ⟨E⟩. In contrast, the shift from position 472 to 475 cannot be resolved reliably. Overall, the FRET efficiency shifts predicted based on the RLA distance distributions are larger than those found experimentally. The AV simulations give results very similar to the RLA simulations for these smaller shifts (cf. Fig. S12; Table S3), and both simulation methods show no relevant difference upon exchange of the two fluorophores. Therefore, the most likely cause for the discrepancies between model and experiment is different local environments of the labeling sites, whose influence on the photophysics or translational and rotational diffusivity of the dyes is not taken into account in the simulations. Our results suggest that the benchmark we have chosen is close to the limits of distance variations that can be resolved by single-molecule FRET.

In the DEER experiments (Figs. 2 and 5 *C*), the expected shifts of the mean distances are clearly visible in all cases except 392/468 → 472. Yet, in both experimental and simulated distance distributions, the shifts are smaller than the widths of the distributions. On average, the FWHM is 0.97 nm, whereas the theoretical distance change for 392/468 → 472 is 0.25 nm. Evidently, this distance change is close to the resolution limit accessible for nitroxide spin labels at solvent-exposed helical sites.

The simulated average distances from the RLA distance distributions exhibit a remarkably high correlation between spin and fluorescence labels (Figs. 5 *D* and S14). Although the widths of the distributions are clearly larger for the fluorescence labels (Tables S1 and S3), the correlation suggests that the average positions of the labels relative to the protein backbone in these cases are surprisingly similar for both types of labels. In contrast, the backbone *C*_*α*_*-C*_*α*_ distances (Fig. S14) reveal a clear offset from the average interlabel distances. Because all analyzed distances are between the same two *α*-helices, the offset happens to be constant here, which is not generally the case. This discrepancy emphasizes the benefit of taking the labels into account.

## Discussion

In various applications, experimental distance distribution constraints between spin labels together with rotamer library simulations have contributed to determining structures or structural models of biomolecules or their complexes by EPR (16,57,113, 114, 115, 116, 117, 118, 119, 120). Similarly, single-molecule FRET has increasingly been used for modeling biomolecular structures (37,61). To facilitate integrative structure modeling that combines experimental data from both EPR spectroscopy and single-molecule FRET, we established here a common framework for comparing structural models to experimental long-range distance constraints from both techniques. The common challenge when applying these techniques to structure determination is that distances are measured between the active centers of the site-specifically incorporated labels: either the unpaired electrons for EPR, or the transition dipoles of the fluorophores for FRET. Hence, to compare the experimental distance constraints with structures, simulations of the labels at the respective protein sites are required. To have a common simulation technique for both types of labels, we have here transferred the RLA simulations, which are capable of predicting even anisotropic spatial distributions (46,56), to fluorescence labels.

In generating the rotamer libraries, we overcame the sampling problem that grows exponentially with the number of flexible dihedral angles in the linker and found that despite the much longer linkers and thus larger number of canonical conformations of the fluorescence labels, representative sets of not more than 1024 rotamers are sufficient for the analysis (Fig. S10). The likely cause of this finding is that the distributions are relatively smooth for the flexible labels, and hence, fewer discrete points become sufficiently representative. In comparison to the established AV approach (58), we found the predictions by RLA simulations to be slightly more precise. This comes at the cost of increased computational effort, but once the rotamer libraries are available, RLA simulations are still possible within seconds on a desktop computer. This is efficient enough for screening large numbers of models, as required for integrative structure modeling.

Comparing the uncertainty in the constraints derived from EPR spectroscopy and single-molecule FRET, i.e., the deviations of the RLA simulations from the experimental results, we find these to be considerably larger in terms of absolute distances for the chromophores. There are several reasons for these uncertainties, some of which we have included in our model. An important contribution is the uncertainty in the Förster radius *R*_0_. Furthermore, protein and fluorophore dynamics make absolute distances more challenging to predict for FRET than for EPR. The simplest approach for FRET uses the static limit (see Results), i.e., it neglects translational diffusion, which causes fluctuations in the interdye distance on a timescale comparable to the fluorescence lifetime, while at the same time the orientation factor is approximated by *κ*^2^ ≈ 2/3 because of the fast rotational diffusion of the chromophores. To include dynamics in the model, we use the simulated distance distributions for obtaining potentials of mean force and include the relative translational diffusion of the dyes on the timescale of the donor-excited state lifetime in the analysis. Our calculations (Fig. 4) show that taking fluorophore dynamics into account has a clear influence when evaluating how well a given model fits a set of constraints. The better the Förster radius *R*_0_ and the diffusion constant *D* can be ascertained experimentally, the more the resulting uncertainties will be reduced until dye quenching and sticking dominate as sources of uncertainty. Sometimes, the latter two effects can be reduced by selecting favorable labeling positions. Despite these uncertainties that give rise to the rather large intervals of *D* and *R*_0_ (Fig. 4), we found that the extent of overlap of the predicted and experimental transfer efficiencies is distinctly different for the different libraries and hence, we were able to select an optimal set of rotamer libraries (in terms of *f* factor, see Fig. S11). The *f* factor determined here is likely to be similar for other dyes with similar linkers that lead to a comparable extent of effective compaction of the linkers and fluorophores, which is affected by the application of the optimal *f* factor in the rotamer library generation (Fig. S10). The generalizability of the optimal *f* factor would ideally be tested for additional proteins. However, because the amino acid composition of the protein used here is not unusual, it is reasonable to assume that similar values of *f* will provide a good approximation also for other folded proteins.

Although the RLA approach provides good agreement with experimental FRET efficiencies on average, the individual values differ from the prediction by <0.025 in half of the cases, and permuting the donor and acceptor results in average differences in the FRET efficiency of 0.04. These deviations are similar to the errors for independent FRET measurements in multiple laboratories obtained in a recent large-scale study (86), suggesting that we are approaching limits in terms of feasible accuracy and precision. Factors that remain untreated in our model, as in most other models, are specific interactions of protein residues with the labels that influence their conformations or, most importantly for FRET, the photophysical properties or orientational distributions of the fluorophores. Accounting for these effects would require a much more detailed treatment with a residue-specific interaction potential. Although the latter is feasible in specifically optimized all-atom force fields (72), aspects such as changes in dye photophysics are beyond current reach. Nevertheless, at this level of accuracy, the predictions are sufficient for quantitative use in integrative structure modeling if the remaining uncertainties are taken into account (86). The approach presented here might work particularly well for modeling conformational changes, in which the local environment at the labeling sites remains the same so that variability in quantum yields and steric restrictions is minimal. If the distance change to be observed is small, site-selective labeling for FRET will be advantageous to avoid averaging over slightly different transfer efficiencies as observed here for the permutants. Hence, the high sensitivity for distance differences we observed here for both EPR and FRET can best be exploited for modeling conformational changes.

Systematic integration of FRET and EPR restraints would be valuable, e.g., because FRET can be carried out at physiological temperatures and can thus be used to test whether and to what extent the conformations of biomolecules are affected by the shock freezing that is required for EPR distance measurements. Conversely, the combination of the two techniques can potentially reveal whether an ensemble of structures derived from the more accurate EPR restraints is representative of the state present in solution, which is accessible with FRET. Using the RLA approach both for EPR and FRET also makes it easier to relate the detailed shape of distance distributions obtained in EPR to the specific distances and dynamics detected by FRET at ambient temperatures. Because both techniques are capable of detecting the mean label-to-label distances, RLA can also help correlate them to the protein backbone *C*_*α*_-*C*_*α*_ distances and evaluate the offset between label-to-label and *C*_*α*_-*C*_*α*_ distances. For the solvent-exposed *α*-helical sites used here, the mean label-to-label distances are very similar for both label types, despite the significant difference in length between fluorescent and paramagnetic labels (Figs. 5 *D* and S14), as also observed previously (121). The origin of this similarity is that the spatial distributions of both fluorescence and spin labels are sufficiently uniform such that the centroids are close to each other for the two types of labels (Fig. S14). Most likely, this is not a general result, and deviations between the two RLA-based mean distances for sites with partial steric hindrance or higher anisotropy of the rotamer distributions around the attachment sites could be larger.

## Conclusions

Although it is challenging to use FRET and EPR for revealing small distance variations on the order of one or two turns of an *α*-helix, we showed here that this resolution could be achieved in most of the cases we probed. This result demonstrates the sensitivity of constraints based on FRET and EPR experiments and RLA for detecting small structural changes for integrative structure modeling. The rotamer library approach could be transferred from spin labels to the significantly larger fluorescence labels. We found that despite the large number of potential rotamers of the fluorescent labels with their long linkers, the approach is computationally feasible because reduced-size libraries of only a few thousand rotamers reproduce the experimental FRET efficiencies well, especially when combined with diffusional averaging that takes the translational motion of the two dyes into account. Using rotamer libraries for both spin and fluorescence labels thus provides a promising perspective for future applications in integrative structure modeling and enhances the synergy between the two complementary methods.

## Author contributions

D.K., A.H., C.G., D.N., M.Y., F.H.-T.A., B.S., and G.J. designed the research. C.G. and I.R. prepared protein samples and carried out EPR experiments. A.H. carried out fluorescence labeling and single-molecule FRET experiments, analyzed by A.H. and D.N. N.B. conducted NMR experiments and analysis. D.K. and G.J. extended MMM with FRET functionality. D.K. generated rotamer libraries and carried out all simulations, analyzed the data, and wrote the manuscript with contributions from all authors.

## Supporting material

Document S1. Supporting materials and methods, Figs. S1–S14, and Tables S1–S3

Document S2. Article plus supporting material
