# Supplementary material for: Resolving distance variations by single-molecule FRET and EPR spectroscopy using rotamer libraries
Source: Biophys J. 2021 Sep 16;120(21):4842–58. doi: 10.1016/j.bpj.2021.09.021 (PMC8595751; doi:10.1016/j.bpj.2021.09.021)
Supplement: Document S1. Supporting materials and methods, Figs. S1–S14, and Tables S1–S3 [file mmc1.pdf]

Biophysical Journal, Volume *120*

Supplemental information

Resolving distance variations by single-molecule FRET and EPR spectroscopy using rotamer libraries

Daniel Klose, Andrea Holla, Christoph Gmeiner, Daniel Nettels, Irina Ritsch, Nadja Bross, Maxim Yulikov, Frédéric H.-T. Allain, Benjamin Schuler, and Gunnar Jeschke

Biophysical *Journal*
Article

Supporting Information: Resolving small distance variations by single-molecule FRET & EPR spectroscopy using rotamer libraries

Daniel Klose[1,*], Andrea Holla[2], Christoph Gmeiner[1], Daniel Nettels[2], Irina Ritsch[1], Nadja Bross[5], Maxim Yulikov[1], Frédéric H.-T. Allain[3], Benjamin Schuler[2,4,*], and Gunnar Jeschke[1]

[1]Department of Chemistry and Applied Biosciences, ETH Zurich, Zurich, Switzerland
[2]Department of Biochemistry, University of Zurich, Zurich, Switzerland
[3]Institue of Biochemistry, ETH Zurich, Zurich, Switzerland
[4]Department of Physics, University of Zurich, Zurich, Switzerland
[5]Department of Chemistry, University of Zurich, Zurich, Switzerland
[*]Correspondence: daniel.klose@phys.chem.ethz.ch or schuler@bioc.uzh.ch

SI METHODS

Structure of CF660R maleimide

The chemical structure of CF660R maleimide (Fig. 1) is based on patent US2012/0329068A1 (Biotium, Inc., Hayward, CA, US) (107). Its mass (1010.18 Da) is within experimental error of the value obtained by mass spectrometry (1011.34 Da). The structure was further confirmed by NMR spectroscopy based on the following experiments: ^{1}H; ^{13}C{^{1}H}; ^{1}H,^{1}H-DQF-COSY; ^{1}H,^{1}H-TOCSY; ^{1}H,^{1}H-ROESY; ^{1}H,^{13}C-HSQC; ^{1}H,^{13}C-HSQC-edited and ^{1}H,^{13}C-HMBC.

RLA simulations of fluorescence labels

The general workflow for the rotamer analysis and calculations of distance distributions is illustrated in Fig. S5 (bottom half) and proceeds in analogy to spin labeling described in the manual of MMM. The steps used here are the following for the two fluorescence labels Cy3b and CF660R that are abbreviated in MMM (Version 2021.1, available as an open source Matlab program on *www.epr.ethz.ch* and on *GitHub.com/gjeschke* (incl. source code to generate rotamer libraries)) by the three-letter codes Cy3 and CF6.

- Load a structure in PDB format into MMM by *File* → *New from PDB/local*.
- Select residues to be labeled by label 1 by *Display* → *Hierarchy*.
- Calculate rotamer populations for label 1 by *FRET* → *Chromophore site scan*. Leave default options in *Site scan setup*. In *Set labeling conditions* select the chromophore library for label 1, here either *Cy3b_2048* or *CF660R_2048*. This selects the default libraries with 2048 rotamers. The rotamer libraries are provided in sizes of N_{rot} = 1024, 2048, 4096 and 8192 (downloaded automatically upon first use). After confirmation the rotamer search for label 1 is performed.
- Select residues to be labeled by label 2 by *Display* → *Hierarchy*.
- Calculate rotamer populations for label 2 by *FRET* → *Chromophore site scan* as above.
- Calculate distance distributions by *FRET* → *Distance distribution/FRET efficiency*. Select two labels one by one and click "+" after each. The distance distribution between the two selected labels will be calculated and shown automatically.
- After having selected two labels the distance distribution and static FRET efficiencies will be calculated. Diffusional averaging with different parameters can be turned on in the bottom left panel.
- The results, i.e. the distance distribution and the FRET efficiencies, can be saved in ascii format using the *Save* button.

In MMM there is a distinction of first calculating rotamer populations and later (optional) attachment, which allows rotamer populations to be calculated for multiple labels at close distances without leading to clashes between the labels. Hence only after attachment, labels are "present" in the structure and therefore considered in clash tests. Here this attachment feature is not used as chromophore pairs are far enough apart not to overlap, however for visualization of the label distributions on the protein structure prior attachment of the labels is required.

AV simulations

AV simulations were carried out using the program FPS 1.1 (58) from the group of Prof. Claus Seidel (University of Düsseldorf, Germany). The required parameters for each label are the linker length l measured from C_β to the center of the chromophore and linker width w as well as three radii that describe the chromophore as an ellipsoid. The parameters were chosen according to the manual of the software using DFT-optimized structures with elongated linkers. For maleimido-Cy3b, this lead to l = 18.5 Å, as well as 3.4, 8.2 and 3.0 Å for the three radii, respectively. For maleimido-CF660R, we derived l = 17.6 Å, as well as 8.1, 4.2 and 2.1 Å for the three radii, respectively. The linker width w was set to 4.5 Å in both cases, which is the same as for other maleimide-functionalized fluorescence labels.

Since in FPS only a single structure is processed from an input PDB file, we loaded the single RRM3/4-ΔN structures, where atoms beyond C_β (attachment point) of the side chains to be labeled had been removed. The resulting AVs for each label (in .xyz file format) were evaluated both via FPS as well as by custom-written Matlab scripts.

SI TABLES AND FIGURES

Table S1: **Summary of the distance distributions of spin labels.** Experimental values from DEER are colored in red. RLA simulations are given in black. r_{cog} is the center of gravity distance, r_{max} indicates the maximum of the distance distributions and FWHM is the full width at half maximum. λ is the DEER modulation depth and "Lab.eff." denotes the spin labeling efficiency according to cw EPR (gray).

**Positions**	**EPR**				
	r_{cog} [a] [nm]	r_{max} [nm]	FWHM [nm]	λ	Lab.eff. [b]
388/468	4.97 | 5.21	5.06 | 5.25	0.87 | 0.89	0.42	67%
388/472	4.71 | 4.86	4.79 | 4.95	1.14 | 0.79	0.26	66%
388/475	4.43 | 4.62	4.50 | 4.70	0.62 | 0.83	0.37	78%
392/468	4.35 | 4.71	4.48 | 5.05	1.06 | 1.11	0.29	62%
392/472	4.33 | 4.46	4.50 | 4.65	0.90 | 1.15	0.35	100%
392/475	3.89 | 4.11	3.92 | 4.50	0.60 | 1.07	0.45	77%

[a] Center of gravity of the upper 75% of the distributions to avoid influence from baseline.

[b] Variations in labeling efficiency are partly also due to residual amounts of unbound spin label (see Fig. S1), which does not contribute to the modulation depth in DEER.

Table S2: **Summary of the distance distributions of fluorescence labels from RLA and AV simulations.** RLA simulations were conducted either using all 20 structures of the NMR ensemble (PDB: 2ADC) (shown in black) or using RRM3/4-ΔN (shown in gray). The strong similarity of the results shows that for distance distributions between fluorescence labels the entire ensemble is well-represented by the RRM3/4-ΔN structure. AV simulations carried out for RRM3/4-ΔN (shown in gray). r_{cog} is the center of gravity distance, r_{max} indicates the maximum of the distance distributions and FWHM is the full width at half maximum.

**Positions**	**FRET - RLA**			**FRET - AV**[b]			
**+Labels**	r_{cog}[a] [nm]	r_{max} [nm]	FWHM [nm]	r_{cog}[a] [nm]	r_{max} [nm]	FWHM [nm]	R_{mp}[c] [nm]
388-A/468-D	5.11 | 5.11	5.20 | 5.20	1.99 | 2.03	5.53	5.60	1.87	5.29
388-D/468-A	5.12 | 5.13	5.20 | 5.20	2.10 | 2.14	5.53	5.55	1.88	5.30
388-A/472-D	4.82 | 4.79	4.90 | 4.80	2.01 | 2.05	5.23	5.20	1.85	4.99
388-D/472-A	4.82 | 4.82	4.90 | 4.90	2.01 | 2.05	5.22	5.40	1.88	5.00
388-A/475-D	4.60 | 4.63	4.75 | 4.70	2.04 | 2.03	4.99	5.10	1.98	4.76
388-D/475-A	4.60 | 4.64	4.70 | 4.75	2.09 | 2.14	4.99	5.20	1.99	4.76
392-A/468-D	4.66 | 4.66	4.75 | 4.70	2.05 | 2.06	5.05	5.15	1.95	4.81
392-D/468-A	4.66 | 4.64	4.75 | 4.70	2.23 | 2.20	5.07	5.10	1.93	4.82
392-A/472-D	4.41 | 4.36	4.50 | 4.40	2.01 | 2.04	4.77	4.80	1.84	4.51
392-D/472-A	4.40 | 4.35	4.50 | 4.50	2.13 | 2.11	4.78	4.80	1.87	4.53
392-A/475-D	4.10 | 4.11	4.20 | 4.20	2.10 | 2.05	4.42	4.50	1.98	4.17
392-D/475-A	4.09 | 4.08	4.20 | 4.20	2.25 | 2.23	4.44	4.60	2.00	4.18

[a] Center of gravity of the upper 75% of the distributions to avoid influence from baseline.
[b] AV simulations carried out on RRM3/4-ΔN.
[c] Midpoint distance between fluorophores from output of AV simulation program FPS.

Table S3: **Summary of FRET efficiencies from experiments and simulations.** Experimental mean transfer efficiencies $\langle E \rangle$ from smFRET, the standard deviation $\sigma(\langle E \rangle)$ from repeated experiments and stoichiometry $\langle S \rangle$. Simulated transfer efficiencies were calculated from distance distributions for both RLA and AV simulations by dynamical averaging (see methods) using an excited donor lifetime of 2.7 ns, a diffusion constant for the interdye distance of $D = 0.2$ nm^2/ns and a Förster radius of $R_0 = 6.0$ nm (FRET-RLA, FRET-AV). The standard deviation in these cases is obtained by variation of R_0 by ±7%, an error estimate reported by a multi-laboratory comparison study (86), and D is varied in the interval $[0\ 2D]$. For comparison, the transfer efficiency E from the AV simulations is given when evaluated with the FPS software (AV-FPS), which uses the static approximation of the Förster equation (58).

**Positions**	**exp. smFRET**			**FRET - RLA**[a]		**FRET - AV**[a]		AV - FPS[a]
**+Labels**	$\langle E \rangle$	$\sigma(\langle E \rangle)$	$\langle S \rangle$	$\langle E \rangle$	$\sigma(\langle E \rangle)$	$\langle E \rangle$	$\sigma(\langle E \rangle)$	E
388-A/468-D	0.745	0.008	0.524	0.728	0.044	0.643	0.052	0.614
388-D/468-A	0.780	0.007	0.492	0.724	0.044	0.643	0.052	0.612
388-A/472-D	0.788	0.006	0.517	0.789	0.037	0.709	0.047	0.680
388-D/472-A	0.824	0.007	0.485	0.785	0.038	0.708	0.047	0.678
388-A/475-D	0.784	0.005	0.522	0.815	0.034	0.754	0.042	0.727
388-D/475-A	0.836	0.006	0.484	0.814	0.034	0.753	0.042	0.726
392-A/468-D	0.792	0.004	0.520	0.808	0.034	0.741	0.043	0.714
392-D/468-A	0.829	0.003	0.496	0.811	0.034	0.738	0.043	0.711
392-A/472-D	0.831	0.005	0.517	0.855	0.028	0.796	0.037	0.771
392-D/472-A	0.880	0.004	0.497	0.857	0.027	0.793	0.037	0.770
392-A/475-D	0.831	0.004	0.520	0.886	0.023	0.848	0.029	0.829
392-D/475-A	0.867	0.002	0.485	0.889	0.022	0.845	0.029	0.826

[a] Simulations carried out using RRM3/4-ΔN.

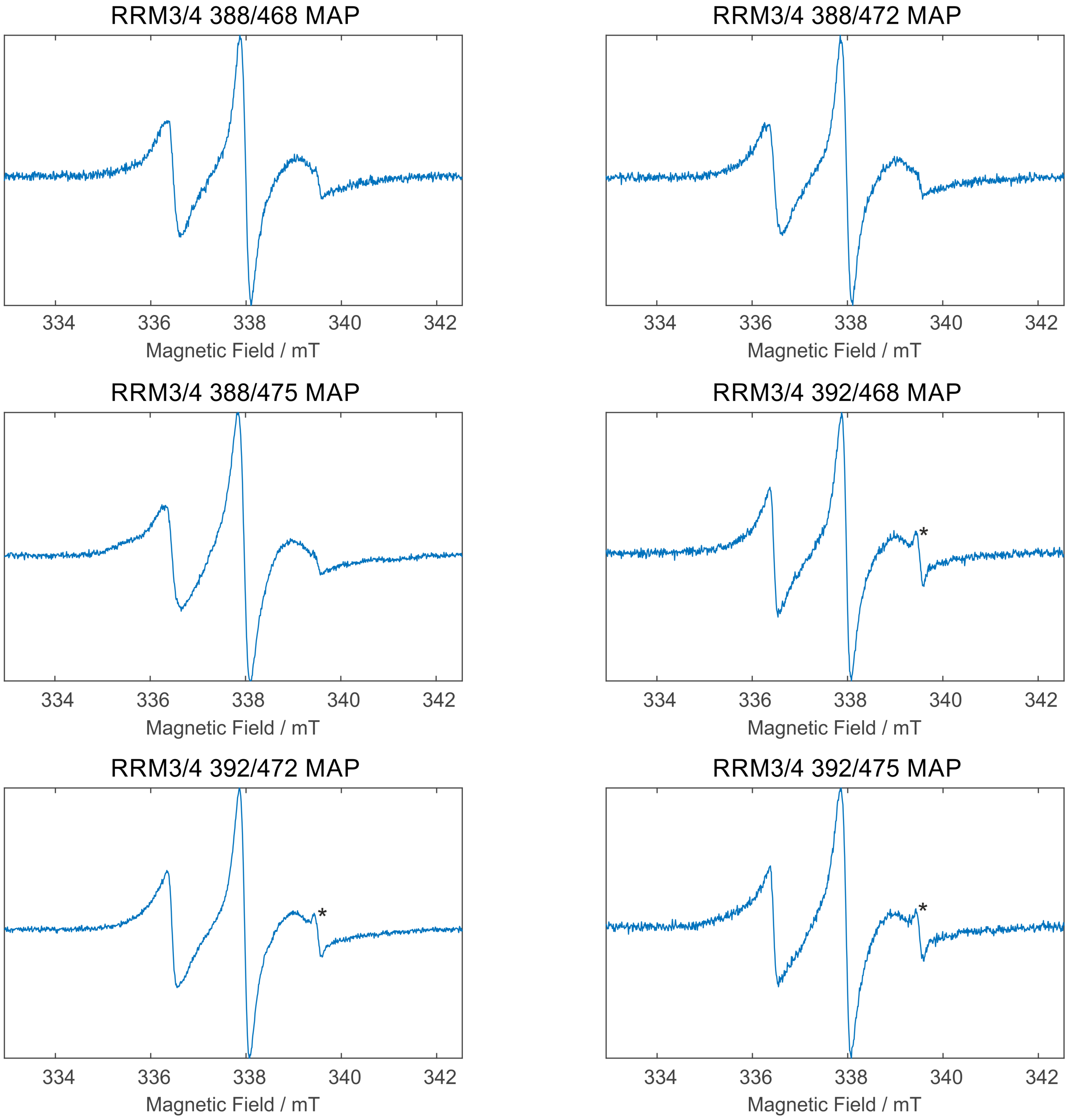

Figure S1: **Verifying spin labeling by cw EPR spectroscopy.** Cw EPR spectra at 9.5 GHz, room temperature, show the amount of bound MAP spin label on RRM3/4. The labeling efficiency determined by double integration is given in Tab. S1. (The asterisks mark putative contributions of residual free label.)

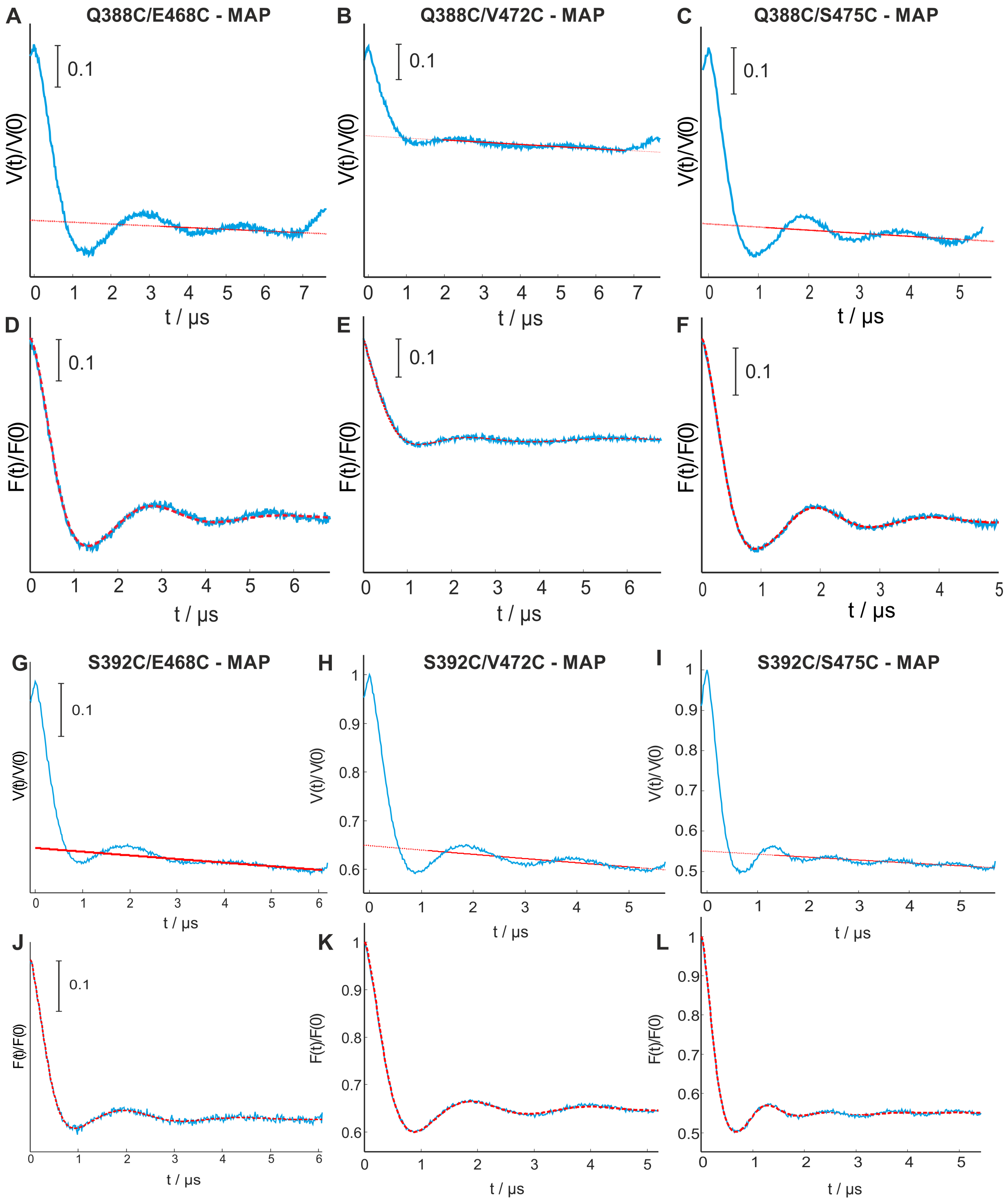

Figure S2: **EPR distance determination by DEER. A-C & G-I** Experimental DEER time traces (normalized) with 3D-homogeneous background fits (red). Cysteine positions in RRM3/4 spin-labeled with maleimido proxyl are given in the legend. **D-E & J-L** DEER form factors (time traces divided by background) with fits (red) that correspond to the distance distributions shown in Fig. 2A. Distance distributions were determined by Tikhonov regularization with regularization parameters determined by the L-curve criterion in DeerAnalysis2016.

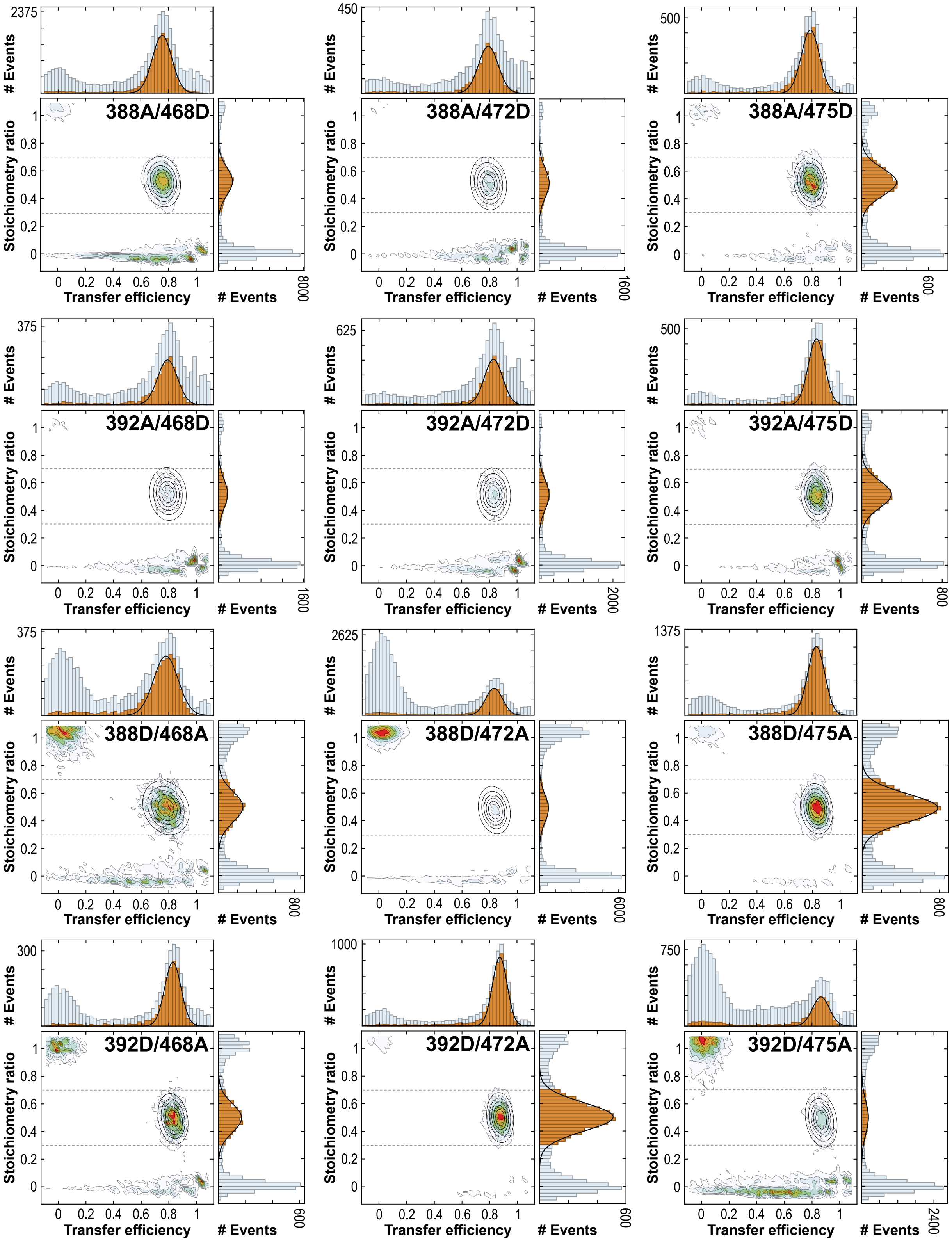

Figure S3: **Single-molecule FRET 2D histograms.** 2D histograms of transfer efficiencies (*E*) versus stoichiometry ratios (*S*) from single-molecule FRET. *S* allows donor-only and acceptor-only subpopulations (top left and bottom of each histogram, respectively) to be separated from donor-/acceptor-labeled sample ($0.3 < S < 0.7$). Labeling positions are shown in the histograms, with D for donor (Cy3b) and A for acceptor (CF660R). Projections along both dimensions show histograms of the full dataset (gray) and the stoichiometry-selected donor-acceptor labeled subpopulations (orange), with selection bounds shown as dashed lines in the 2D histograms.

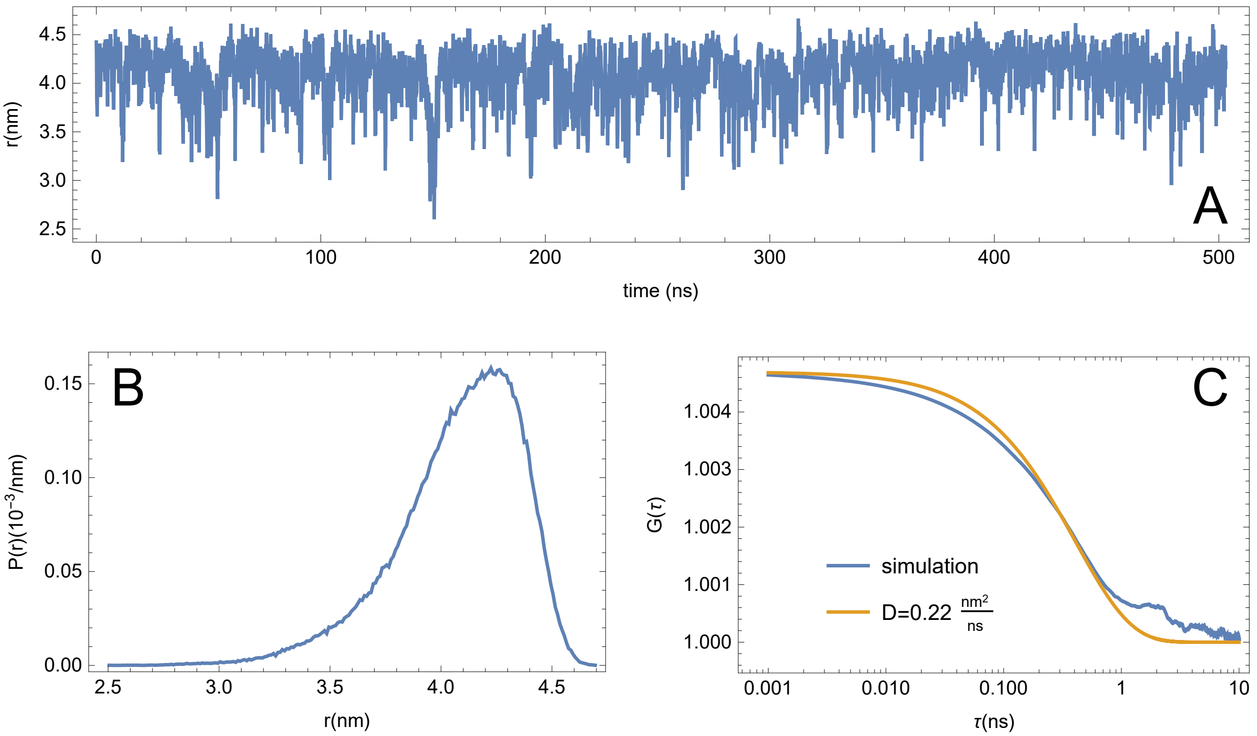

Figure S4: **Inter-dye distance time-trajectory** from an all-atom MD simulation of polyproline-11 labeled via an N-terminal Gly with Alexa594 and via a C-terminal Cys with Alexa488-C5-maleimide (72). **A** Fluctuation of the inter-dye distance r over the time course of 500 ns.**B** Distance distribution $P(r)$ obtained from the trajectory shown in A. **C** Time-correlation of $r(t)$ calculated from the trajectory shown in A (blue). The yellow correlation curve is calculated from Eq. 9 with $P(r)$ from B assuming an effective diffusion coefficient of $D = 0.22\ \text{nm}^2/\text{ns}$. A single diffusion coefficient is not sufficient for fully describing the correlation curve obtained from the simulation, which comprises a major fast component (~ 0.3 ns) and a minor slow component (~ 3 ns). However, both timescales are comparable to the lifetime of the excited state of the donor (4 ns in the absence of the acceptor).

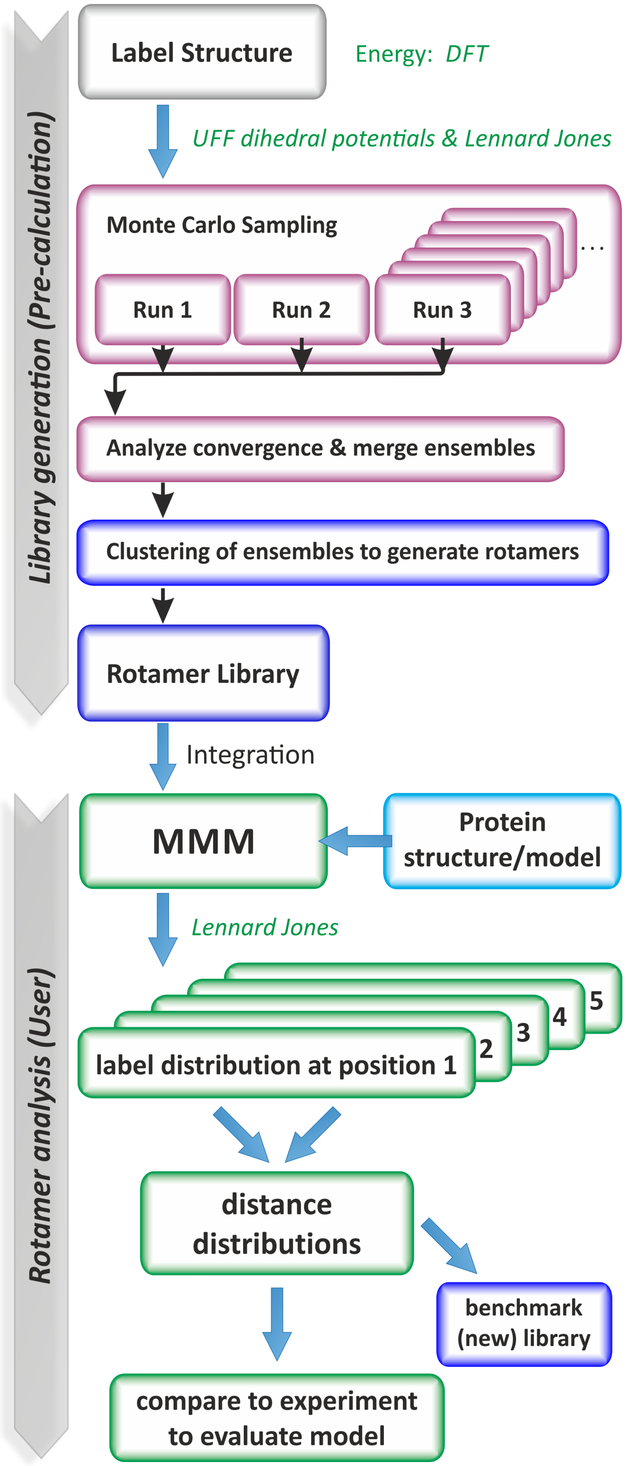

Figure S5: **Flowchart for rotamer library generation and rotamer analysis of a protein structure.** Generation of a rotamer library *(top half)* for a specific spin or fluorescence label is carried out in three stages *(color-coded)*. First, a minimum-energy 3D structure of the label is obtained by DFT calculations. Second, the full label conformational space is sampled by Monte Carlo using rotatable dihedral angles as degrees of freedom. Third, clustering of the Monte Carlo ensemble leads to discrete rotamers. These stages are done once, and the resulting library is integrated into MMM for further use. Calculating distance distributions between labeling sites *(bottom half)*. For each labeling site in a given protein structure or model, the conformational distribution of the label is calculated using the pre-calculated rotamer library. For any pair of labeling sites, a distance distribution is then calculated as a weighted histogram comprising all possible combinations of label conformations at the two sites. The distance distributions can be benchmarked against experimental data for a known system to validate new rotamer libraries *(as in this study for Cy3b & CF660R)* and then used with any structure for analysis and modeling.

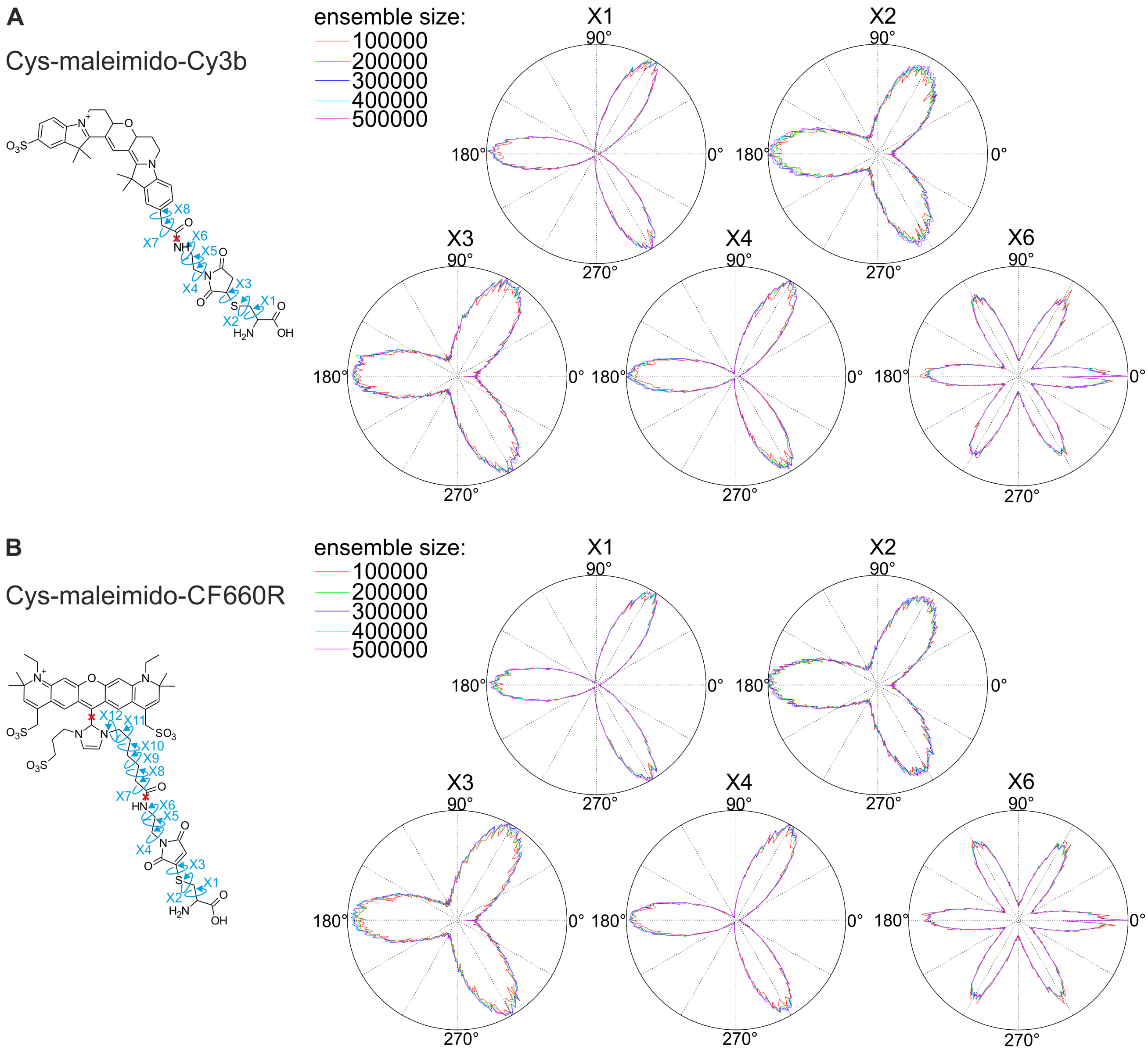

Figure S6: **Dihedral angles and convergence of Monte Carlo Sampling.** Definition of dihedral angle numbering (left) and dihedral angle histograms (right) for Monte Carlo ensembles of different sizes (see legend) for Cy3b and CF660R labeled side chains in **A** and **B**, respectively. The histograms show that the dihedral angle distributions still contain significant noise due to undersampling at an ensemble size of 100000, while there is barely any change observed in the histograms between ensemble sizes of 400000 and 500000, indicating the onset of convergence.

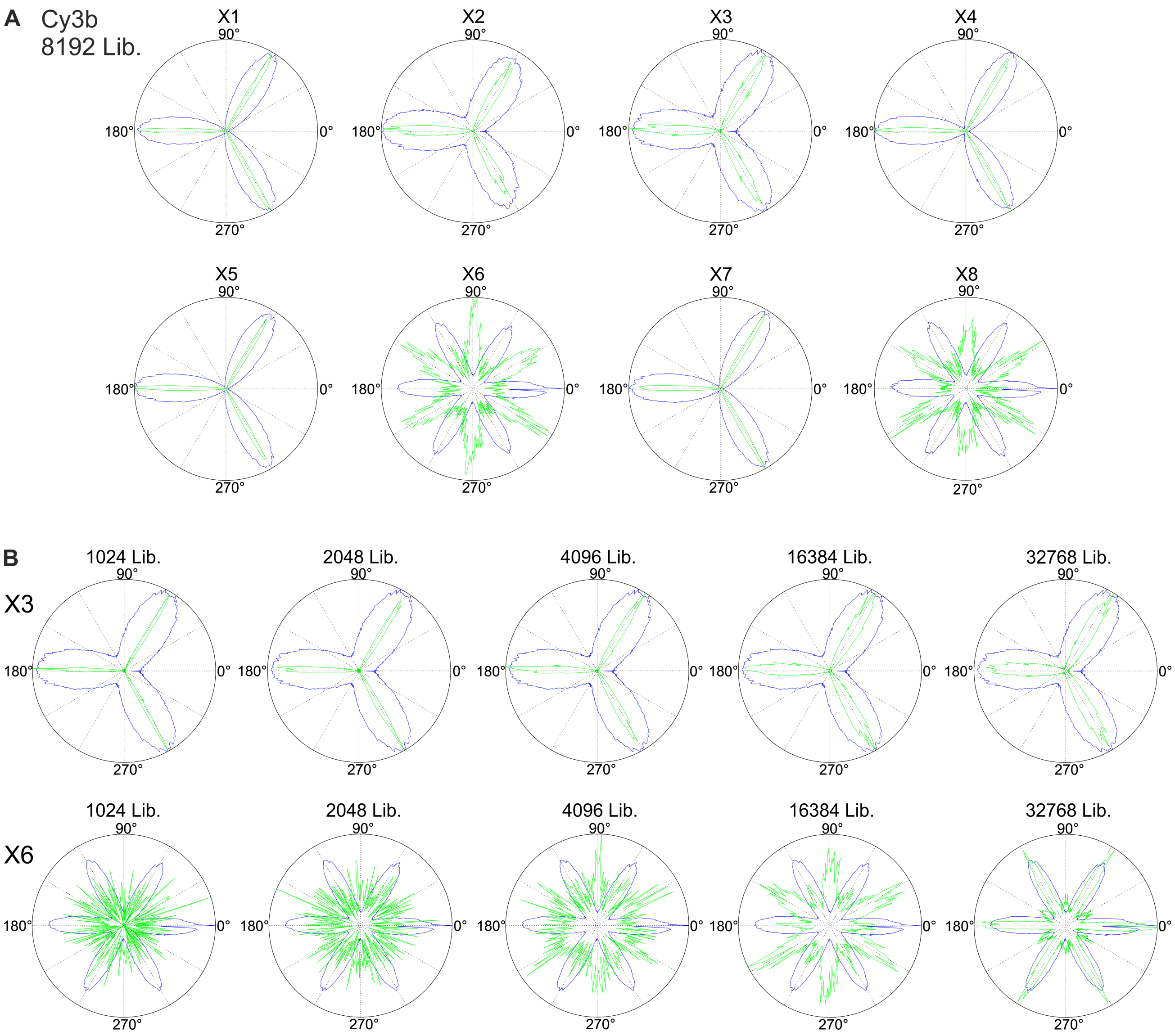

Figure S7: **Rotamer library generation: Dihedral sampling of Cy3b.** Dihedral angle histograms of Cy3b-labeled side chain (eight rotatable bonds with dihedrals X1-X8). The 500,000 member Monte Carlo ensemble is shown in blue, rotamer libraries in green. **(A)** Histograms of dihedral angles X1 to X8 for a rotamer library size of 8192. **(B)** Histograms of the two dihedral angles X3 and X6 (representative of the threefold and sixfold dihedrals, respectively) for increasing rotamer library sizes from 1024 (left) up to 32768 rotamers (right). The histograms show that dihedrals featuring three-fold rotation symmetry are well-represented in the rotamer libraries, albeit dihedrals are closer to the canonical values in the rotamer libraries versus the Monte Carlo ensemble. In contrast, for the two dihedrals X6 (and X8), strongly limited library sizes lead to averaging across different canonical dihedral angles, which leads to pronounced populations in between the canonical dihedral angles up to 8192 rotamers, while for the largest library, which is sufficiently sized to cover all canonical angle combinations for the eight dihedrals, this effect is almost absent. This effect was, however, found to have no significant influence on the resulting distance distributions calculated using different rotamer library sizes (see Fig. S10).

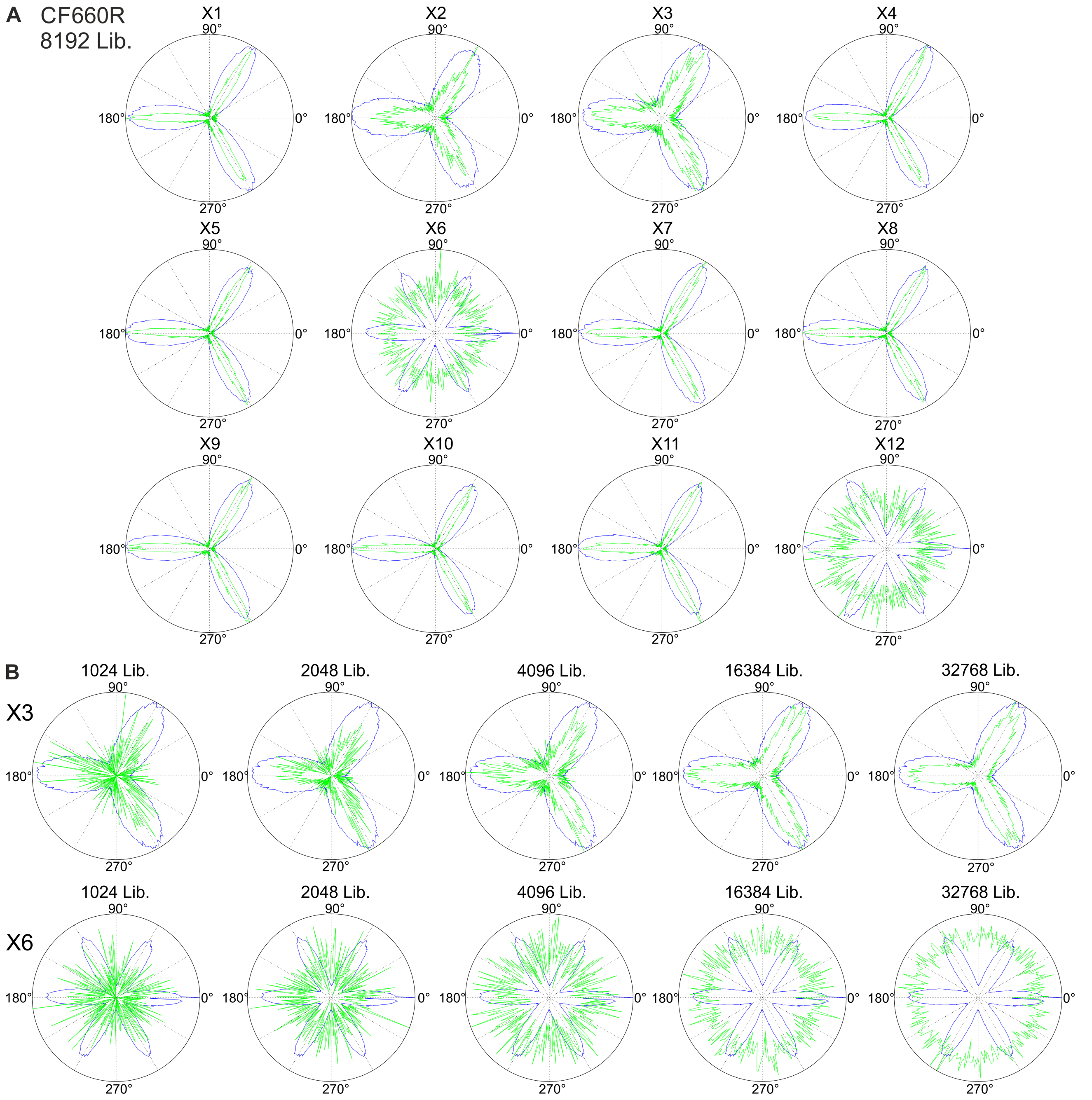

Figure S8: **Rotamer library generation: Dihedral sampling of CF660R.** Dihedral angle histograms of CF660R-labeled side chain (twelve rotatable bonds with dihedrals X1-X12). The 500,000 member Monte Carlo ensemble is shown in blue, rotamer libraries in green. **(A)** Histograms of dihedral angles X1 to X12 for a rotamer library size of 8192. **(B)** Histograms of the two dihedral angles X3 and X6 (representative of the threefold and sixfold dihedrals, respectively) for increasing rotamer library sizes from 1024 (left) up to 32768 rotamers (right). The histograms show that dihedrals featuring three-fold rotation symmetry are well-represented in the rotamer libraries, albeit dihedrals are closer to the canonical values in the rotamer libraries versus the Monte Carlo ensemble. In contrast, for the two dihedrals X6 (and X12), strongly limited library sizes lead to averaging across different canonical dihedral angles, which leads to pronounced populations in between the canonical dihedral angles even for the largest library of 32768 rotamers. This effect was, however, found to have no significant influence on the resulting distance distributions calculated using different rotamer library sizes (see Fig. S10).

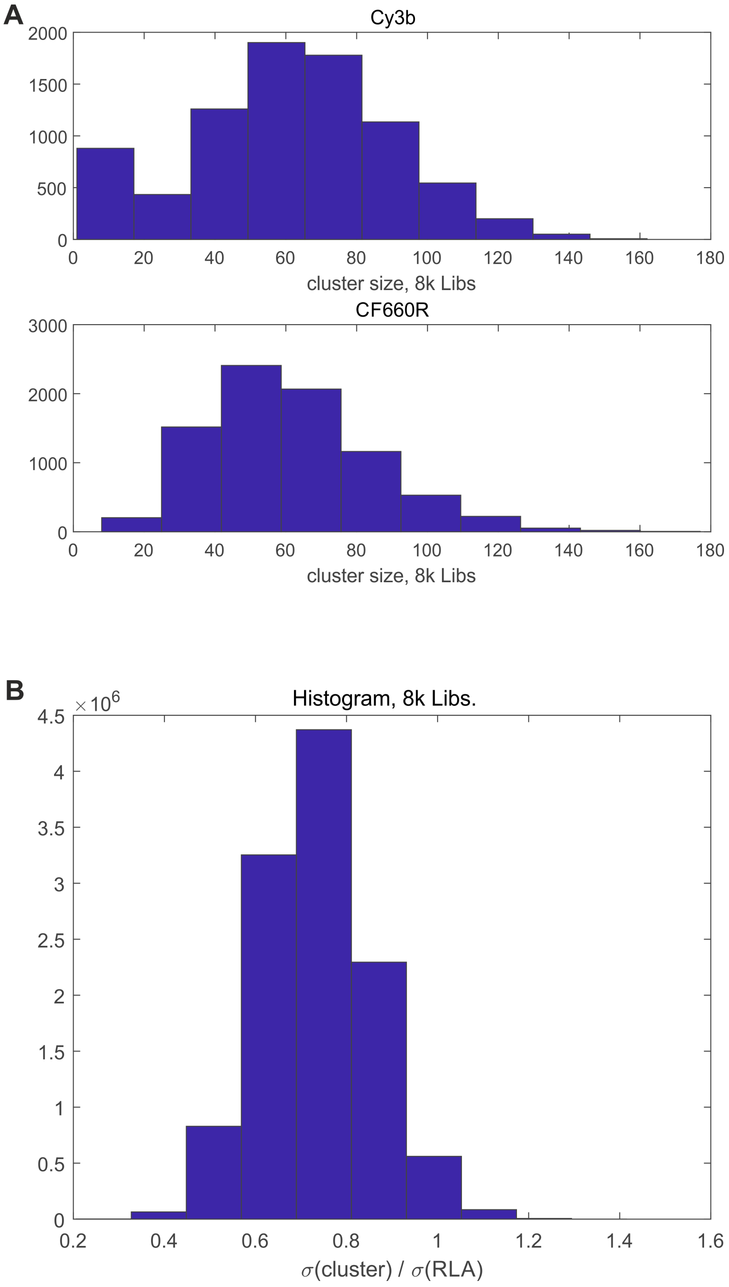

Figure S9: **Clustering test for the fluorescence label rotamer libraries. A** Size distributions of the clusters that give rise to one rotamer for a library of size of 8192 for Cy3b (top) and CF660R (bottom). **B** Distance distributions between pairs of clusters for a library size of 8192 were calculated for coordinates corresponding to labeling positions 388-Cy3b/475-CF660R on RRM3/4 (without clash tests and accordingly with uniform weights), and the standard deviation σ(cluster) obtained for each pair. The standard deviation of the distance distribution between the two full Monte Carlo ensembles σ(MC) = 0.943 nm is obtained by random sampling from the two ensembles. The histogram of the relative widths σ(cluster)/σ(MC) peaks at ca. 0.75 showing that the many distributions between two clusters are slightly narrower than the distribution between the full ensembles. This ratio becomes smaller at larger library sizes. Due to the heavy computation required these calculations were carried out to convergence rather than exhaustively.

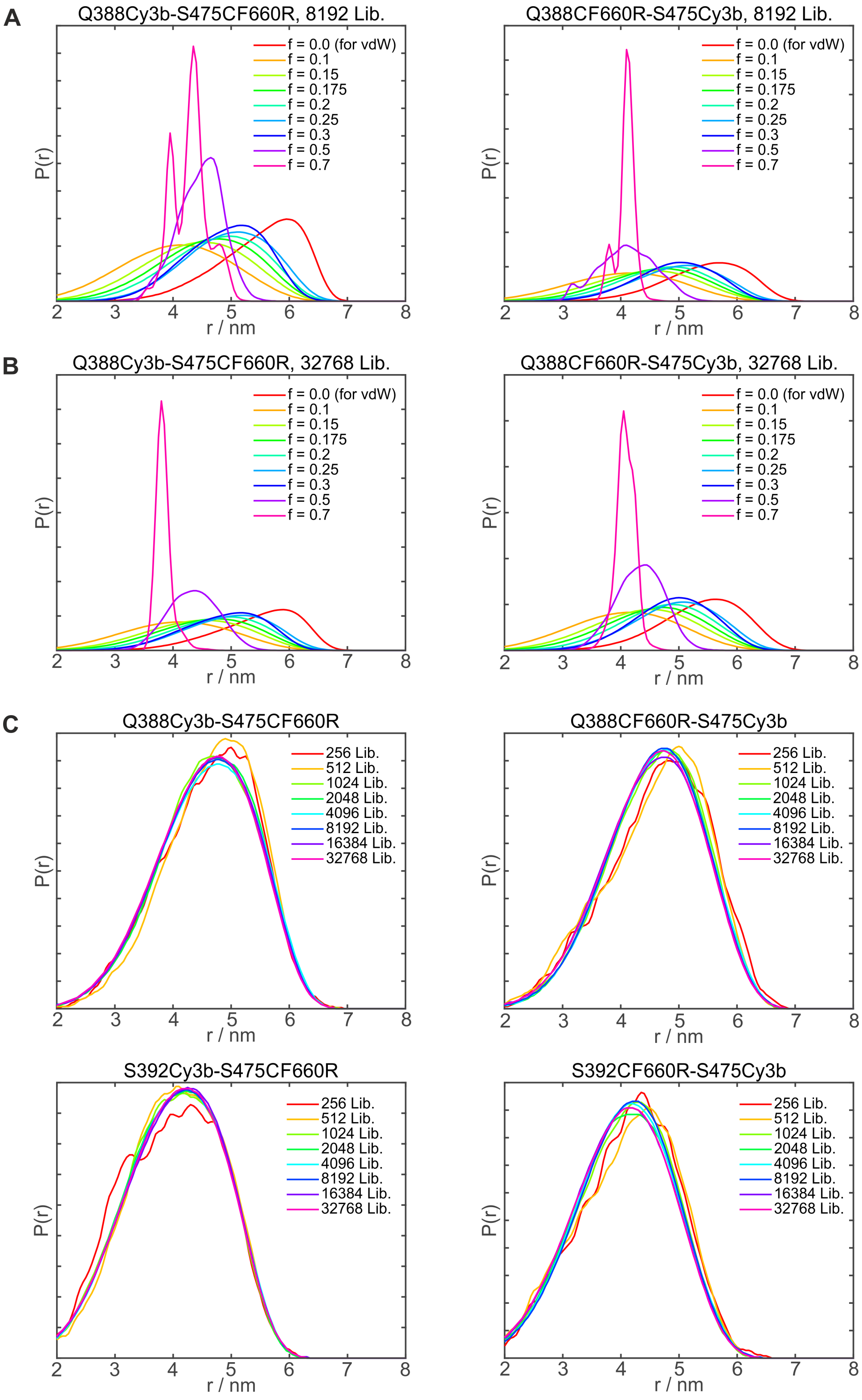

Figure S10: **Distance distributions between fluorescence labels from rotamer analysis: Testing for f factor and library size.** Representative distance distributions of RRM3/4 (PDB 2ASW, structure1-ΔN-term.) using different f factors (see legend) with rotamer library sizes of 8192 and 32768 rotamers in **A** and **B**, respectively. The distributions show a complex dependence on the f factor and indicate that libraries with a larger f factor require larger sizes for convergence with these labels. Where $f = 0$ for the van der Waals interaction, for the repulsive interaction f is kept at 0.7 for maximum contrast (cf. Eq. 1). **C** Representative distance distributions using the optimal f factor of $f = 0.175$ (see Fig. S11) using different rotamer library sizes (see legend). The strong similarity of the distributions shows that at this low f factor, small rotamer libraries (with respect to the number of canonical rotamers) of 1024 to 2048 rotamers yield results with sufficient sampling.

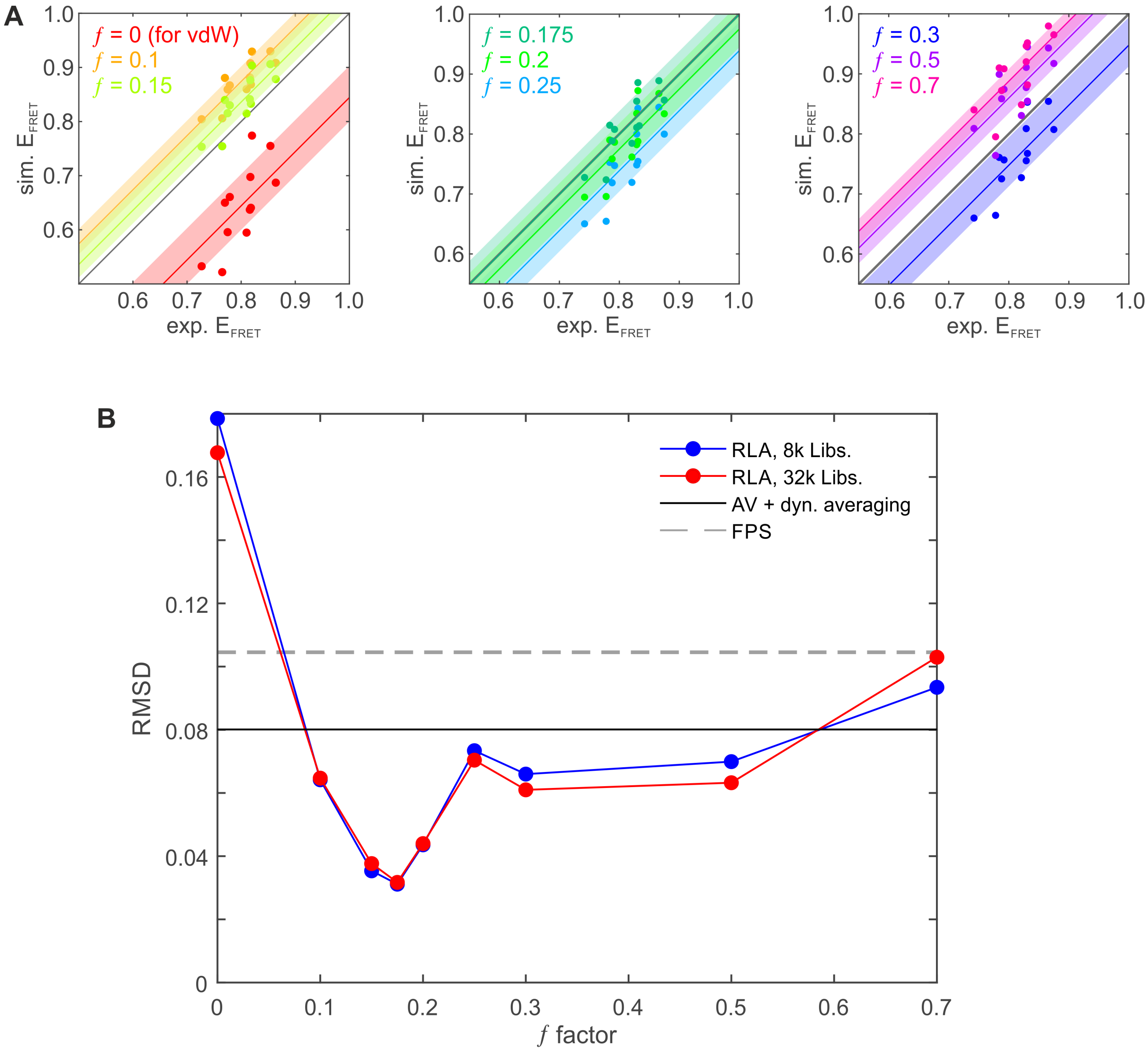

Figure S11: **Choice of f factor for the rotamer libraries - RMSD values vs. experiments. A** Experimental versus simulated FRET efficiencies. Simulated FRET efficiencies (points) from diffusional averaging (D = 0.2 nm^2/ns and R_0 = 6 nm) using RLA libraries with different f factors, color-coded according to the legend. Standard deviations of simulated FRET efficiencies from twelve RRM3/4 variants due to uncertainties in Förster radius R_0 and translational diffusion constant D are indicated by color-shaded areas. Linear fits with slope 1 to the simulated FRET efficiencies (colored lines) emphasize the deviation from ideal 1:1 correlation (gray line). Note that the influence of the d ifferent f factors is larger than that of R_0 and D (given by the color-shaded areas), such that the different f factors can be distinguished to identify the optimum value of f. Further note, where f = 0 for the van der Waals interaction, for the repulsive interaction f is kept at 0.7 for maximum contrast (cf. Eq. 1). **B** RMSD values of experimental versus simulated FRET efficiencies for all f factors for 8k and 32k rotamer library sizes (blue and red, respectively) and AV distance distributions (see Fig. S12) after diffusional FRET averaging assuming D = 0.2 nm^2/ns (black), as well as transfer efficiencies from the AV approach simulated with the FPS software (gray), where the inter-dye distance is assumed to be static on the timescale of the excited-state lifetime of the donor. Since lower RMSD values represent better agreement with the experimental results, we obtain f = 0.175 as the optimal f factor.

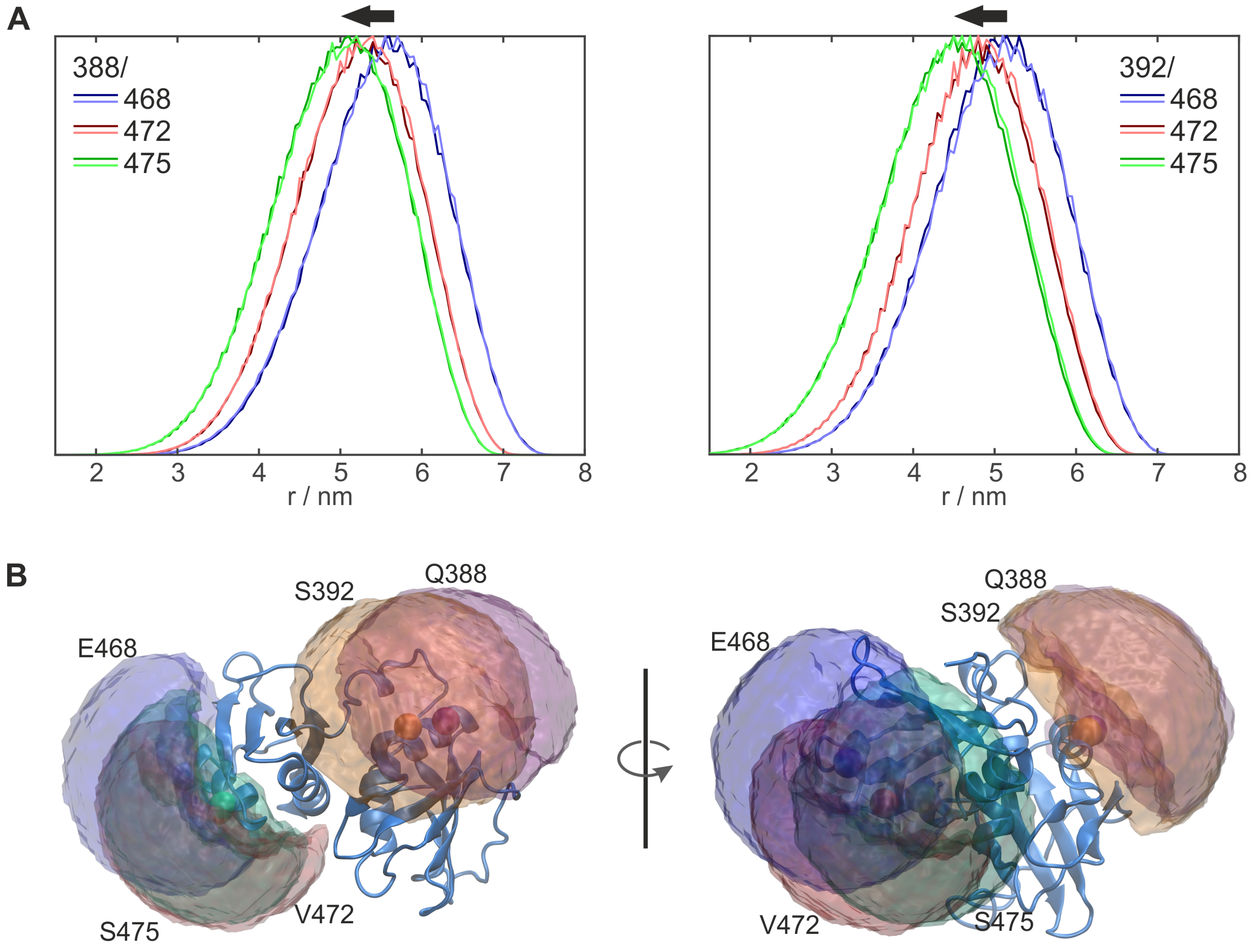

Figure S12: **AV simulations: Distance distributions and spatial distributions. A** Distance distributions calculated from AV simulations as a histogram over all pairwise distances between voxels of two accessible volumes. The labeling positions are indicated by color (see legend), with dark colors indicating the acceptor CF660R and light colors indicating the donor Cy3b at positions Q388C (left) and S392C (right). Arrows symbolize the shifts to smaller distances from E468C (blue) to S475C (green). **B** RRM3/4-ΔN (blue ribbons, PDB: 2ADC with N-terminal truncation) with accessible volumes of Cy3b at positions Q388C and S392C, and CF660R at positions E468C, V472C and S475C. From left to right, the structure is turned in the direction indicated by about 45°.

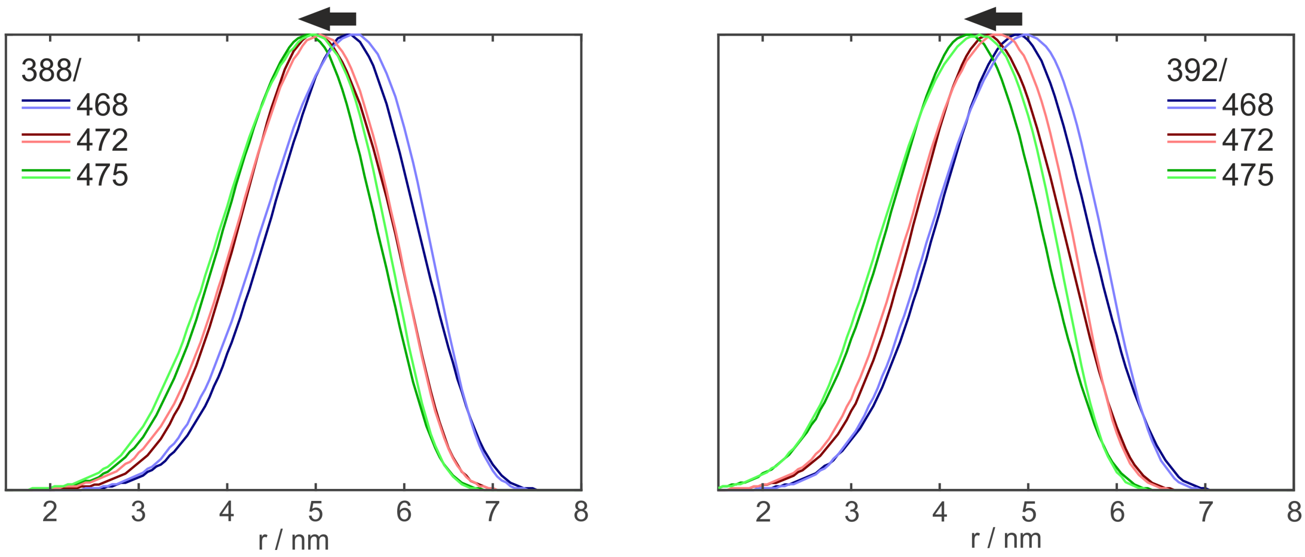

Figure S13: **Distance distributions between fluorescence labels calculated by RLA simulations for RRM3/4-ΔN.** RLA distance distributions based on the structure of RRM3/4-ΔN (PDB: 2ADC, first structure with N-terminal truncation). The labeling positions are indicated by color (see legend), with dark colors indicating the acceptor CF660R and light colors indicating the donor Cy3b at positions Q388C (left) and S392C (right). Arrows symbolize the shifts to smaller distances from E468C (blue) to S475C (green). These calculated distance distributions are virtually unchanged upon exchange of donor and acceptor fluorophores (see also Fig. 3 and Fig. 5 for corresponding FRET efficiencies), and are insensitive to the presence of the flexible N-terminus in the ensemble simulations (Fig. 3) *versus* the simulations based on RRM3/4-ΔN (here).

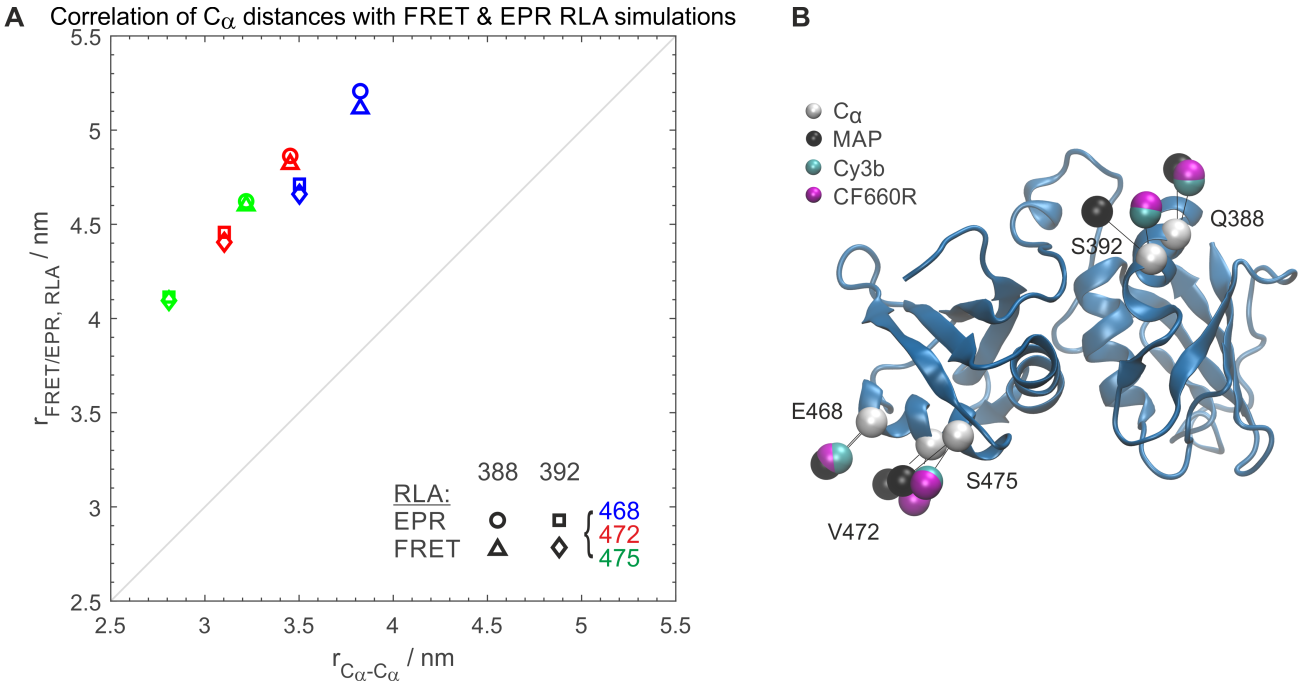

Figure S14: **Backbone Distances *versus* label-label distances. A** Correlation of backbone C_α-C_α distances to distances between labels determined by the RLA center of gravity distances for EPR and FRET (see Tabs. S1 and S2 for data). **B** 3D visualization of spin and fluorescence label midpoints (population-weighted mean of spatial distribution) on RRM3/4 (ribbon representation). Spheres represent the C_α atoms and the label midpoints according to the color code in the legend. The midpoints of spin and fluorescence labels on RRM3/4 show an RMSD of 2.1 Å.
